# Pervaporative Dehydration of 2,3-Butanediol by Dense Poly(vinylidene fluoride) Hollow Fiber Membranes: Parameter Estimation, Process Design, and Technoeconomic Evaluation under Uncertainty

**DOI:** 10.1021/acs.iecr.5c01198

**Published:** 2025-08-12

**Authors:** Marco Avendano, Blake Trusty, Shailesh Dangwal, Zachary Coin, Syed Islam, Sankar Nair, Ramesh Bhave, Matthew J. Realff

**Affiliations:** † School of Chemical and Biomolecular Engineering, 1372 Georgia Institute of Technology, Atlanta, Georgia 30332, United States; ‡ Oak Ridge National Laboratory, Chemical Sciences Division, Oak Ridge, Tennessee 37830, United States

## Abstract

Pervaporation, combined with other separation processes, can effectively remove water from fermentation product streams, making it highly suitable for purifying alcohols like 2,3-butanediol (BDO). In this study, a dense poly­(vinylidene fluoride) (PVDF) hollow fiber membrane module prototype was fabricated for BDO dehydration, achieving >0.2 LMH total flux and >95% BDO rejection. With a Markov chain Monte Carlo (MCMC) approach, Bayesian inference was used to quantify the uncertainty of the permeance parameters. A membrane cascade model was developed to scale up a process that purifies a preconcentrated BDO feed (70 wt %) to high purity (90 wt %). Through propagation of the uncertainty of the parameters and sensitivity analyses of the process variables, a cascade design was recommended. Despite data and model limitations, the framework enabled a reliable system analysis and economic evaluation, validated through tight confidence intervals in key process metrics, establishing the foundation for future applications of Bayesian methods in membrane-based processes.

## Graphical abstract

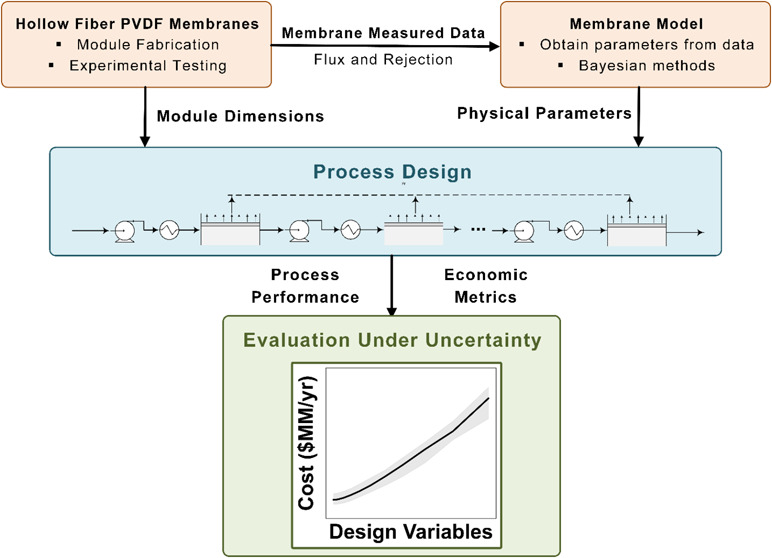

## Introduction

Pervaporation membranes have the potential to substantially reduce costs and emissions of critical industrial-scale applications, since it combines membrane permeation and evaporation in a single unit.^1^ This process is presented conceptually in Figure S1, where the mixture of components flows along the surface of the membrane on the upstream (or feed/retentate) side while the main permeating component is continuously rejected to the downstream (or permeate) side via vaporization. The combination of permeation and evaporation selectivity can overcome thermodynamic constraints such as azeotrope formation or increase in the conversion rate of equilibrium-limited reactions by continuous separation of the product.^2–5^ The mechanism of pervaporation consists of three steps: (1) sorption at the membrane surface on the feed/retentate side, (2) diffusion through the membrane, and (3) desorption under vacuum conditions at the surface on the permeate side.^6^ This setup enables the interaction of liquid and gas phases without direct contact, with the membrane serving as a selective boundary. Pervaporation is most effective when the membrane selectively permeates the undesired minority component in the feed stream, making it ideal for “polishing” separations in which the feed has already been preconcentrated and a further high purity is required.^7^ Depending on the nature of the membrane material (hydrophilic or organophilic), pervaporation can be used to remove organics and pollutants from wastewater,^8^ separate organic mixtures with similar boiling points,^9^ and dehydrate concentrated organic streams.^10^ However, membrane performance (flux and rejection) for these challenging mixtures are highly dependent on experimental operating conditions, and only a few studies have developed process models to address the practical and economic impact of pervaporation membranes on a large scale.^3,11–13^

Pervaporation has strong potential in biorefineries,^14^ where fermentation typically yields organic products at low titer concentrations, and hybrid separation processes are needed to produce high-purity streams. A notable example is the production of high-purity ethanol (>99%) from a dilute fermentation product, wherein distillation produces a preconcentrated ethanol/water mixtures near the azeotrope, and pervaporation then breaks the azeotrope by selectively removing water and achieves high purity.^6^ However, with volatile components like ethanol, there are significant losses of the organic species to the permeate. This can result in large recycling streams and increased membrane system size. Heavy alcohols offer a promising opportunity, as they are considerably less volatile than water.^15^ Among such species, 2,3-butanediol (henceforth referred to as “BDO”) has garnered recent interest and has been explored by researchers for recovery using pervaporation membranes. Dettwiler et al. reported the first application of pervaporation to recover BDO through testing over a polyether block amide (PEBAX) film.^16^ A process simulation was developed where a pervaporation membrane was integrated with a bioreactor to continuously remove BDO and acetoin from an aqueous fermentation broth, preventing product inhibition effects and enhancing overall performance.^17^ Qureshi et al. developed a pervaporation membrane distillation (PMD) system using a poly­(tetrafluoroethylene) (PTFE) commercial hollow fiber membrane, which concentrated BDO from 4.25 wt % to 65 wt % in an aqueous mixture.^18^ Fadeev et al.^19^ and Chovau et al.^20^ measured the sorption of BDO on flat sheet poly­[1-(trimethylsilyl)-1-propyne] (PTMSP) and poly­(dimethylsiloxane) (PDMS) membranes, respectively. BDO exhibited high sorption in both studies, but due to its low volatility no BDO concentration was detected in the permeate. More recently, sulfonated polybenzimidazole membranes with graphene oxide additives (sPBI/GO) have been proposed for BDO recovery from dilute mixtures through pervaporation.^21–24^ To demonstrate enrichment capability of sPBI/GO, a 10 wt % BDO aqueous feed was concentrated to 20 wt % through a continuous lab-scale operation over a polymer coated porous ceramic membranes that maintained high separation factors and reasonable flux.^21^ A membrane cascade was then proposed to purify a preconcentrated 30 wt %. BDO stream to 50 wt %.^23^ Required reheating between membrane stages resulted in very high energy demands. It was concluded that pervaporation is likely not suitable for direct BDO preconcentration from high water content feeds, and instead its application was recommended for more concentrated BDO streams. Shao and Kumar explored BDO recovery by combining pervaporation with liquid–liquid extraction using butanol as the solvent.^25–28^ Polymeric and polymer/zeolite composite membrane coupons over a Teflon plate (flat sheets) were tested to recover dilute BDO (<5 wt %) from an organic phase rich in butanol (>85 wt %). They then introduced ZSM-5 fillers in the PDMS membranes to improve separation. A cascade of membranes with interstage recycling was proposed for achieving 99.5 wt % BDO purity at a 75% recovery. Significant energy savings over vacuum membrane distillation were obtained and the authors also evaluated the effect of ethanol on process performance. While the substantial economic impact of low recovery was not assessed, the importance of pervaporation for BDO separation was further illustrated by the study.

In 2018, the National Renewable Energy Laboratory (NREL) established a state-of-technology (SOT) assessment for a biorefinery producing a renewable hydrocarbon fuel blendstock from corn stover, with BDO as the alcohol intermediate.^29^ BDO is obtained at dilute conditions (10 wt %) via fermentation. Due to its much higher boiling point (177 °C) than water, it cannot be economically preconcentrated by distillation due to the large amount of water boilup required. However, a higher BDO purity (>90 wt %) is required for its efficient catalytic conversion of BDO to renewable fuels through a catalytic upgrading train. In recent work, we modeled and experimentally demonstrated a continuous (SMB) adsorption process using MFI-type nanozeolite adsorbents to selectively preconcentrate BDO by rejecting water and other organics (fermentation byproducts, unreacted carbohydrates).^30–32^ This enabled the development and experimental validation of a simulated moving bed (SMB) adsorption process, which we combined with distillation and integrated within the biorefinery design flowsheet. In this modified biorefinery design, the proposed SMB+distillation separation produces a preconcentrated stream with BDO purity >70 wt % at >99.9% recovery. This provides a significant opportunity for the use of pervaporation membrane system to purify the preconcentrated BDO to >90 wt %. Alternative process designs to recover BDO at comparable purity and recovery values have been proposed, including steam-stripping,^33^ extraction followed by distillation,^34–36^ multieffect evaporation,^37^ reactive distillation^38^ and membrane plus extraction.^39^ Compared to these alternatives, pervaporation offers a smaller size footprint, modularity, the possibility of reduced energy and no external substances.

In this study, we aim to develop and demonstrate PVDF hollow fiber pervaporation membranes to selectively remove water from a preconcentrated BDO stream and then develop a model that predicts membrane performance followed by integration into a membrane cascade design. Two main issues are targeted. First, the preparation of hollow fiber membranes and modules is desirable for scalable applications of pervaporation in biorefineries for BDO recovery, departing from the widely used flat sheet/coupon membranes in previous works.^16,19,20,25,26^ Second, pervaporation processes are strongly dependent on intrinsic material properties and face significant uncertainties in prediction and operation. Determining performance parameters and accurately describing membrane behavior is challenging due to limited experimental data, high sensitivity to measurement errors, and scarcity of process models that can reliably predict performance. Prior studies have generally quantified uncertainty in membrane-based processes,^40–46^ yet only a few have focused on pervaporation. Chen et al. derived an expression to predict an additive error term for flux as a function of state variables and applied this approach to isopropyl alcohol purification.^41^ Heitmann et al. evaluated the effect of uncertainty by conducting a sensitivity analysis on permeance parameters.^43^ However, neither study directly quantified the uncertainty of membrane-specific parameters through probability distributions, which is a desirable and more direct approach to provide better insights into the underlying physical mechanisms and their impact on process design.

Based on the above two issues, the goal of this work is to develop a robust framework that combines hollow fiber membrane fabrication, experimental evaluation and data collection, and uncertainty quantification to design and assess a pervaporation-based process for purifying BDO. To address the variability inherent in membrane behavior we propose estimating the uncertainty in the parameters of the dense PVDF hollow fiber module using Bayesian inference, unlike previous pervaporation studies that rely on additive error terms or sensitivity analyses. This comprehensive approach enables a reliable process and technoeconomic foundation for broader applications of such membrane-based separation systems.

## Methodology

### Hollow Fiber Membrane Fabrication

Porous hollow PVDF fibers with mean pore size of 30 nm and Kynar Aquatec ARC solution (PVDF dispersion) were procured from Arkema Inc. while cross-linker Easaqua XD 401 was procured from Vencorex. The length of the fibers was ∼12 in. with inner and outer diameters of ∼0.75 and 0.95 mm, respectively. Porous fibers were pretreated by soaking them in water at 90 °C for 1 h followed by air drying for 1 h at 60 °C. Diluted PVDF dispersion (30 mL PVDF dispersion with 470 mL of DI water) and Easaqua (1 g) were used as the dope solution and cross-linker, respectively for the dense coating. The coating solution was prepared by mixing dilute PVDF dispersion and Easaqua for 30 min on a stir plate stirring at 300 rpm. This solution was used to coat fibers from outside. Each fiber was coated four times by dipping into the solution for ∼5 s followed by 30 min of air drying before subsequent coating. The fibers were dried in air overnight after completion of the membrane coating. Different parameters such as PVDF dispersion dilution, Easaqua amount, and number of coatings were varied to achieve a thin, continuous, and defect free coating. Investigation of the optimal conditions for coating formation as well as SEM images of the fibers before and after various preliminary dip coat conditions can be found in Section S1 of the Supporting Information. After final drying, the fibers were cured at 115 °C for 1.5 h with 1 °C min^–1^ of heating from room temperature (∼1.5 h) and 1 °C min^–1^ of cooling back to room temperature (∼1.5 h). Postcuring, the dense PVDF fibers were used for the membrane module preparation. To prepare the membrane module, two size 8 vinyl mesh screens were punched out at 1 in. diameter using a punch tool and 20 fibers were threaded through one of the mesh screens. The ends of the fibers were carefully bundled together, one just above the screen, and one at the opposite end of the fibers. Then the fibers were guided into the module housing (1″ PVDF tube, fabricated in-house). The nonscreened side was unbundled and threaded through the second screen, and epoxy (Loctite EA 9462 Resin) was used to seal the gap between the mesh screen and top of the module. After overnight drying of one side, the other end was sealed using epoxy. Excess fibers were finally trimmed off from either end of the module after epoxy was dried. The total surface area of one module was approximately 0.018 m^2^. Figure S4 shows the experimental setup used for the pervaporation experiments. We defined total membrane volumetric flux, *J*_LMH_ (L m^–1^h^–1^), and BDO rejection (%) as the metrics to evaluate membrane performance. *J*_LMH_ is measured by collecting the permeate volume over time and normalizing by module surface area and permeation duration. BDO rejection is defined in eq 1 as the fraction of BDO rejected to the retentate and computed with the following expression, where *w*_perm,water_ and *w*_feed,water_ are the weight composition of BDO in the permeate and feed, respectively.

1 $$BDO\;rejection=\left(1-\frac{w_{perm,BDO}}{w_{feed,BDO}}\right)\times 100\%$$

### Quantification of BDO Concentrations

BDO content in both permeate and retentate was done using a Shimadzu LC-2030 Plus with UV detector. The samples were analyzed using InfinityLab Poroshell 120 SB-C18, 4.6 × 150 mm^2^, 4 μ column with corresponding guard column using a mobile phase of 80 wt % methanol in water with 1 mL/min flow rate. The sample injection volume was 1 μL done via autosampler. UV detection was set at 254 nm. Instrument sensitivity could reliably measure BDO content in water down to 0.5 wt %.

### Pervaporation Theory and Modeling

In this study, we selected the well-known solution-diffusion model proposed by Wijmans and Baker^47^ to describe mass transfer through the pervaporation membrane. Equations 2–9 summarize this model. According to the model, the molar flux, *J*_*M,i*_ (mol m^–2^ s^–1^), of each component *i* through the membrane is estimated by computing the product between molar permeance, *Q*_*m,i*_ (mol m^–2^ s^–1^ Pa^–1^), and driving force, DF_*i*_ (Pa).

2 $$J_{M,i}=Q_{m,i}\times {\mathrm{DF}}_{i}$$

3 $$Q_{m,i}=f\left(w_{\mathrm{feed},\mathrm{water}},T_{\mathrm{feed}};\theta_{i}\right)$$

4 $${DF}_{i}={\mathrm{f̂}}_{feed,i}^{G,hypoth}-{\mathrm{f̂}}_{perm,i}^{G}={\mathrm{f̂}}_{feed,i}^{L}-{\mathrm{f̂}}_{perm,i}^{G}$$

5 $${\mathrm{f̂}}_{\mathrm{feed},i}^{L}=x_{i}\gamma_{i}P_{\mathrm{sat},i}\Lambda_{i}$$

6 $${\mathrm{f̂}}_{\mathrm{perm},i}^{G}=y_{i}\phi_{i}P_{p}$$

7 $$\sum x_{i}=1$$

8 $$\sum y_{i}=1$$

9 $$J_{M,i}=y_{i}J_{M}$$

Here, *Q*_*m,i*_ captures both sorption and diffusion phenomena within the membrane and is dependent on the feed’s water weight fraction, *w*_feed,water_ (kg kg^–1^), and temperature, *T*_feed_ (*K*). Rather than relying on fully theoretical equations, it is common to use a semiempirical expression, *f*, to obtain the value of permeance as a function of membrane-specific parameters θ_*i*_. As shown by Thiess et al.,^11^ DF_*i*_ can be derived from Fick’s first law as the difference in fugacity between the gas in the permeate side, *f̂*_perm*,i*_^G^, and a hypothetical gas, *f̂*_feed*,i*_^G,hypoth^ that is in equilibrium with the liquid, *f̂*_feed*,i*_^L^, on the feed/retentate side. Molar fraction of the permeate gas is *y*_*i*_ (mol mol^–1^), vacuum pressure is *P*_*p*_ (Pa) and ϕ_*i*_ is the fugacity coefficient, which was assumed to be ∼1. For the liquid, *x*_*i*_ (mol mol^–1^) is the feed molar fraction, γ_*i*_ is the activity coefficient, *P*_sat,*i*_ (Pa) is pure component vapor pressure and Λ_*i*_ is the Poynting correction factor, whose contribution was also assumed to be negligible (Λ_*i*∼1_).

The values of γ_*i*_ for BDO and water were estimated using the UNIQUAC activity coefficient model.^48^ Fitted binary interaction parameters for vapor–liquid equilibrium data at 10 kPa were obtained from the DECHEMA series.^49^ *P*_sat_ was estimated using Antoine’s equation, with the constants retrieved from the NIST database.^50^ It was also assumed that the permeate stream is an ideal solution and can be obtained by summing the flux of each individual components, eq 9. The UNIQUAC binary interaction parameters and Antoine equation constants for BDO and water can be found in Section S2 of the Supporting Information.

### Permeance Estimation

Permeance parameters, **θ**, are strongly dependent on membrane-specific properties and can only be calibrated with measured data. As a result, semiempirical Arrhenius-like relations are commonly used to estimate permeance as a function of the main design variables ***X*** (*w*_feed,water_ and *T*_feed_ in this case). Equations 10–14 summarize the proposed permeance expressions.

10 $$Q_{m}=f\left(X;\theta \right)=f\left(w_{feed,water},T_{feed};\theta \right)$$

11 $$f\left(w_{feed,water},T_{feed};\theta \right)=Q_{0}\times \exp\left(A_{\mathrm{con}}\times w_{feed,water}+\frac{E_{a}}{R}\times \left(\frac{1}{T_{ref}}-\frac{1}{T_{feed}}\right)\right)$$

12 $$E_{a}=E_{\mathrm{diff}}+{\Delta H}_{\mathrm{sorp}}$$

13 $$\theta ={\left[{\left\{\theta_{i}\right\}}_{i=1}^{N_{\mathrm{comp}}},T_{\mathrm{ref}}\right]}^{T}={\left[\theta_{\mathrm{BDO}},\theta_{\mathrm{water}},T_{\mathrm{ref}}\right]}^{T}$$

14 $$\theta_{i}={\left[Q_{0,i},A_{\mathrm{conc},i},E_{a,i}\right]}^{T}$$

The dependence of permeance on temperature is defined by the apparent activation energy, *E*_a_ (J mol^–1^), which is a function of two competing factors: *E*_diff_ (J mol^–1^), activation energy of diffusion, and Δ*H*_sorp_ (J mol^–1^), enthalpy of sorption.^2^ While increasing temperature enhances diffusion (*E*_diff_ > 0), sorption is an exothermic process (Δ*H*_sorp_ < 0) and decreases with temperature. *E*_A_ captures the net effect of diffusion and sorption within the membrane and in the context of an Arrhenius behavior can provide a physical interpretation of the collected data.^13^ The relation with feed composition is less understood and to reflect the influence of water composition on permeance, researchers often add an empirical term characterized by a proportionally constant, *A*_conc_.^12,13,51–53^ Lastly, *Q*_0*,i*_ (mol m^–2^ s^–1^ Pa^–1^) is the pre-exponential permeance term, *T*_ref_ (K) is a reference temperature and *R* is the universal gas constant (8.314 J mol^–1^ K^–1^). As proposed by Pereira et al.,^51^ eq 10 represents the most general form of the permeance expression. For two components (BDO and water), this results in seven calibration parameters as shown in eq 15.

15 $$\theta ={\left[Q_{0,\mathrm{BDO}},A_{\mathrm{conc},\mathrm{BDO}},E_{A,\mathrm{BDO}},Q_{0,\mathrm{water}},A_{\mathrm{conc},\mathrm{water}},E_{A,\mathrm{water}},T_{\mathrm{ref}}\right]}^{T}$$

Given the uncertainty of both the experimental data and the proposed model, is often preferable to simplify the expression and reduce the number of calibration parameters. In practice, this can be achieved by fixing certain elements of **θ** to constant values, including zero. This effectively reduces the size of **θ** (number of degrees of freedom), minimizing risk of overfitting and improving the robustness in the estimation of the remaining parameters.

### Membrane Performance Prediction Given Experimental Data

Given *w*_feed,water_ and *T*_feed_, the values of Q_*m,i*_, γ_*i*_ and *P*_sat,*i*_ can be computed for each component. This is all the information needed to predict *J*_*M*_ and *y*_*i*_ by solving the nonlinear system of (eqs 2–9) of the model. For only two components, these equations can be arranged into the quadratic form shown in (eqs 16–19) to solve explicitly for *y*_BDO_. *y*_water_ and *J*_*M*_ can then be directly calculated from *y*_BDO_ using (eqs 8 and 9), respectively. The derivation of this expression can be found in Section S4.3

16 $$a\cdot y_{\mathrm{BDO}}^{2}+b\cdot y_{\mathrm{BDO}}+c=0$$

17 $$a=P_{p}\left(Q_{\mathrm{water}}-Q_{\mathrm{BDO}}\right)$$

18 $$b=Q_{\mathrm{BDO}}{\mathrm{f̂}}_{\mathrm{feed},\mathrm{BDO}}^{L}+Q_{\mathrm{water}}{\mathrm{f̂}}_{\mathrm{feed},\mathrm{water}}^{L}+P_{p}\left(Q_{\mathrm{BDO}}-Q_{\mathrm{water}}\right)$$

19 $$c=-Q_{\mathrm{BDO}}{\mathrm{f̂}}_{\mathrm{feed},\mathrm{BDO}}^{L}$$

These are mole-based quantities, which need to be converted to the units *J*_LMH_ (l m^–2^ h^–1^), *w*_perm,BDO_ (kg kg^–1^) and *w*_perm,water_ (kg kg^–1^) for a direct comparison with the measured performance data. To convert from molar to volumetric flux the density of the mixture is needed as a function of feed composition and temperature. A relation for obtaining the mixture’s molar density was developed in Section S2.3 of the Supporting Information. BDO rejection can then be readily estimated using eq 1.

### Bayesian Uncertainty Quantification

The parameter estimation is performed by identifying the set of values that minimize the error between model predictions and experimental measurements. However, this estimation is subject to aleatoric (instrument error) and epistemic (model) uncertainty, the latter arising from the semiempirical nature of the permeance expressions. As the uncertainty carries over to the process, deterministic estimates of **θ** may fail capture the variability and lead to unreliable results.

Thermodynamic parameters (γ_*i*_ and *P*_sat,*i*_) were derived from established methods and references and thus can be treated as deterministic variables in the model. The uncertainty is then attributed entirely to the permeance parameters, **θ**. These are treated as random variables in the model and can be used to propagate the uncertainty of the data to the process design and economic analysis. Bayesian uncertainty quantification provides a systematic approach for addressing this challenge and inferring the unknown joint distribution or posterior density of the parameters, *p*(**θ** *|**Y***), given observed information, ***Y***, (the measured experimental data). Details of the Bayesian framework can be found in Section S3.1 of the Supporting Information.

To estimate the posterior distribution, researchers often employ stochastic sampling techniques, with Markov chain Monte Carlo (MCMC) methods as a popular choice and the Metropolis–Hastings (MH) algorithm as a widely used implementation of these methods. We developed an adaptation of the MH algorithm based on previous studies that also considered material-based separations.^54–58^ Details can be found in Section S3.2 of the Supporting Information.

### Process Design

Figure 1 shows the proposed process design as a cascade of N pervaporation stages, where each stage consists of a feed pump, a preheater and a membrane module. A vacuum system is also required to maintain the required vacuum for all membranes. In pervaporation, the latent heat needed to evaporate the permeant is provided by the sensible heat of the feed. As a result, the temperature drops along the length of the membrane as the permeate stream is continuously being produced. This decreases saturation pressure in the feed/retentate side and reduces fugacity to the point that the driving force and flux eventually become zero. Maintaining isothermal conditions throughout the entire membrane is often impractical and additional stages with constant reheating of the retentate between modules is required to sustain the pervaporation. When considering the overall design, the feed to the first stage is the stream leaving the proposed SMB+distillation BDO preconcentration step. Rather than representing the entire fermentation broth with all components, we considered a simplified binary feed containing only BDO and water. The high BDO purity exit stream is the retentate leaving the last stage of the cascade, which is routed to the BDO upgrading step. The permeate produced by each module is collected in a composite permeate stream that is condensed before being sent to the wastewater treatment area of the biorefinery. A single condenser is located upstream of the vacuum pump to minimize the vapor load to the vacuum system.

**Figure 1:**
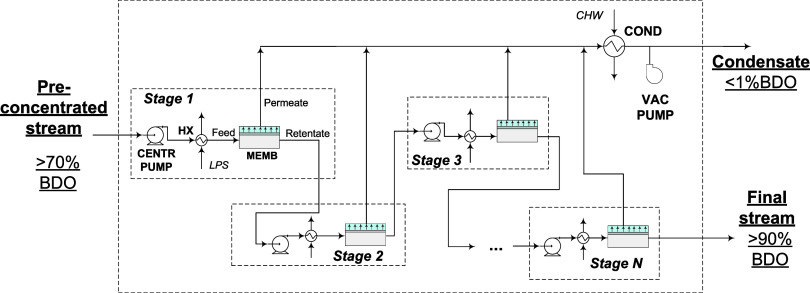
Proposed cascade of N pervaporation stages to obtain a high purity (>90 wt %) BDO stream from a preconcentrated (>70 wt %) feed obtained from an SMB + distillation step. Each stage consists of a feed centrifugal pump (CENTR PUMP), preheater (HX), and membrane module (MEMB). The system also includes a general condenser (COND) and vacuum pump (VAC PUMP) to collect the permeate as a low-BDO (<1 wt %) condensate. Heat is provided by low-pressure steam (LPS) and cooling by chilled water (CHW).

The governing expressions shown in (eqs 20–22) were derived by considering the mass and energy balances around a differential-area element of a membrane module. As shown in Figure 2, we assumed a crossflow pattern, where the liquid in the feed/retentate side travels along the surface of the membrane, while the vapor in the permeate side flows perpendicularly away from it. The local vapor streams produced along the membrane eventually mix to form a single bulk permeate stream. Since the flow rate is small and the pressure is low, it is reasonable to assume that the permeate vapor can be instantly swept away by the vacuum collection medium. This prevents any build-up on the permeate surface and spatial variations can be neglected on this side of the membrane. We assumed plug-flow, no axial dispersion, negligible film effects, steady-state and adiabatic conditions. The details of the derivations can be found in Section S.4.1 of the Supporting Information. The following are the obtained differential equations, where membrane area *A* (m^2^) is used as the space variable. For each module *j*, *ṅ* ^*j*^ is the molar flow rate (mol s^–1^), *x*_*i*_^*j*^ is the liquid mole fraction of component *i* (mol mol^–1^) and *T* ^*j*^ is temperature (K). *J*_*M*_^*j*^ and *y*_*i*_^*j*^ are still molar flux and mole composition of the permeate, respectively, and *T*_perm_^*j*^ is the temperature in the permeate side (K). *Ĉ*_*p*_ is specific molar heat capacity at constant pressure (J mol^–1^ K^–1^) and Δ*H* ^vap^ is molar heat of vaporization (J mol^–1^).

20 $$\frac{d{ṅ}^{j}}{dA}=J_{M}^{j}$$

21 $$\frac{dx_{i}}{dA}=J_{M}^{j}\frac{y_{i}^{j}-x_{i}^{j}}{{ṅ}^{j}}$$

22 $${ṅ}^{j}\left(\sum_{k}x_{k}^{j}{Ĉ}_{p,k}\right)\frac{dT^{j}}{dA}=\sum_{k}J_{M}^{j}y_{k}^{j}\left[{Ĉ}_{p,k}\left(T_{\mathrm{perm}}^{j}-T^{j}\right)+\Delta H_{k}^{\mathrm{vap}}\right]$$

**Figure 2:**
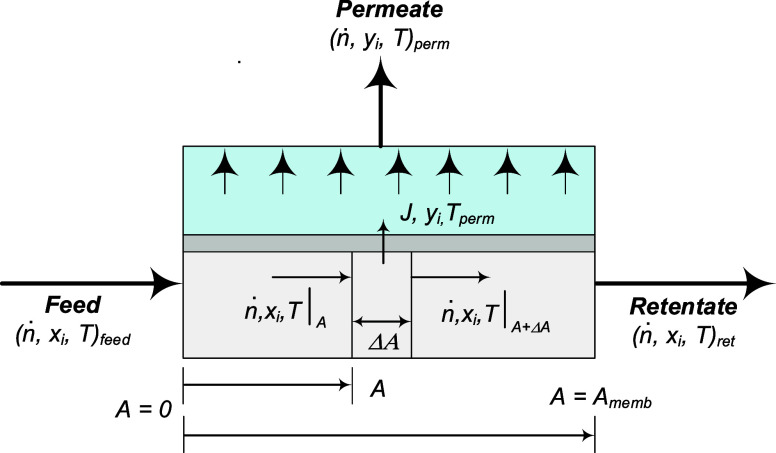
Diagram detailing the state variables of a pervaporation membrane module operating under a tangential crossflow pattern. The govern equations for this module were derived by taking area, ***A***, as the space variable and performing mass and energy balances around a differential-area element (Δ ***A***). The arrows indicate the direction of flow.

As indicated in (eqs 23 and 24), the boundary conditions are only defined for differential state variables, ***z***, which in this case are the variables that characterize the feed/retentate side. Those related to the permeate are instead algebraic state variables, ***v***, which are independent of spatial derivatives due to the well-mixing assumptions of the crossflow model. As mentioned, *J*_*M*_^*j*^ and *y*_*i*_^*j*^ are obtained by solving the quadratic expression in (eqs 16–19), where the permeances, *Q*_*i*_, are estimated at the corresponding values of *x*_*i*_^*j*^ and *T* ^*j*^. Lastly, *T*_perm_^*j*^ is determined by calculating the dew point of the permeate vapor at *P*_*p*_ (Pa), which is the vacuum pressure applied to the system, and is the same for every module.

23 $${ṅ}^{j},x_{i}^{j},T^{j}=z\left(A;\theta \right)$$

24 $$J_{M}^{j},y_{i}^{j},T_{\mathrm{perm}}^{j}=v\left(A;\theta \right)$$

In the cascade design, the retentate of a given module becomes the feed for the next stage and is raised to a certain feed inlet temperature, *T*_inlet_^*j*^ (K). Thus, at the entrance (*A* = 0), the values of *ṅ* ^*j*^ and *x*_*i*_^*j*^ are the same as the outlet of the previous stage (*A* = *A*_memb_^*j*–1^). For the first stage (*j* = 1), the inlet conditions, ***z*** ^0^, correspond to those of the preconcentrated BDO stream. This can be described using (eqs 25–28).

25 $${ṅ}^{j}{|}_{A=0}={ṅ}^{j-1}{|}_{A=A_{\mathrm{memb}}^{j-1}}$$

26 $$x_{i}^{j}{|}_{A=0}=x_{i}^{j-1}{|}_{A=A_{\mathrm{memb}}^{j-1}}$$

27 $$T^{j}{|}_{A=0}=T_{\mathrm{inlet}}^{j}$$

28 $$z^{j=1}{|}_{A=0}=z^{0}$$

These expressions lead to a system of differential algebraic equations (DAE), which can be solved explicitly using Euler’s method. The steps to solve the system of DAEs and design the cascade are detailed in Section S4.2 in the Supporting Information. For simplification, the initial and final BDO concentrations will be fixed throughout this study. The inlet temperature, *T*_inlet_^*j*^ (K), and the maximum allowable membrane area, *A*_memb,max_^*j*^ (m^2^), of each stage will be treated as decisions variables to be further explored via sensitivity analysis. The goal is propagating uncertainty to the process design with permeance parameters as the only random variables in the equations.

### Technoeconomic Analysis

A techno-economic assessment (TEA) was developed based on the mass and energy balances of the resulting cascade design. This includes the purchase equipment costs, *C*_*p*_ ($ U.S dollars), and utilities of the relevant process components. As shown in eq 29, *C*_*p*_ encompasses *N* membrane modules (*C*_*p,modules*_), *N* feed centrifugal pumps (*C*_*p*,feed pump_), *N* preheaters (*C*_*p*,preheater_), one condenser (*C*_p,cond_) and one vacuum pump (*C*_*p*,vacpump_). The feed pumps are centrifugal units and depending on their size (heat transfer area) the heat exchangers (preheaters and condenser) are either shell-and-tube or double-pipe exchangers. For the vacuum system a one-stage sealed liquid-ring pump was assumed, which can realistically achieve pressure values as low as 7 kPa. The sizing and purchase equipment costs expressions were obtained from correlations in Seider et al.^59^ and were adjusted using a Chemical Engineering Plant Cost Index (CEPCI) value of 541.7 for the year 2016 (same as the original biorefinery design^29^).

29 $$C_{p}=C_{p,\mathrm{cond}}+C_{p,\mathrm{vacpump}}+\sum_{k=1}^{N}\left(C_{\mathrm{preheater}}^{k}+C_{p,\mathrm{feedpump}}^{k}+C_{p,\mathrm{modules}}^{k}\right)$$

For the modules, we followed the sizing and cost guidelines outlined by Constantino et al.^3^ Each module is represented by a vessel containing tightly packed membrane fibers arranged in a triangular pitch and the purchase cost can be estimated using eq 30. The cost of the modules was determined based on the estimated membrane area and a unit area cost (*C*_memb_) of $100/m^2^, which typically accounts for the cost of the vessel. This is within the range of membrane unit costs reported in literature.^60–67^

30 $$C_{p,\mathrm{modules}}^{j}=C_{\mathrm{memb}}A_{\mathrm{memb}}^{j}$$

Utilities include low-pressure steam (LPS) for the preheaters, chilled water (CHW) for the condenser and electricity for the pumps. LPS is generated internally in a combined heat and power (CHP) area, with natural gas imported as boiler fuel to meet any additional steam requirements.^29^ CHW is supplied from a refrigeration loop within the biorefinery, and electricity is imported from the grid. In addition, yearly membrane costs were also considered, assuming a frequency of 5 years. This is within the range of membrane lifetime values typically assumed for pervaporation studies. More details regarding the stability and lifetime of the PVDF membranes can be found in Section S5.2 of the Supporting Information.

To determine the total annualized cost (TAC) expression in eq 31, we recognize that the proposed cascade is not a stand-alone system but is integrated within an existing biorefinery plant. We developed an empirical expression for TAC, where heat (MW), cool (MW) and elec (kW), are the LPS, CHW and electricity duties of the system, respectively. The multipliers (*a, b, c, d* and *e*) are thus effective marginal unit costs and their values represent the cost of introducing additional equipment and utilities to the plant. *R* (%) is BDO recovery in the cascade and is used as a penalizing factor since BDO lost to the permeate is sent to wastewater treatment and needs to be compensated by increasing throughput. Details of the equations used to compute heat, cool, elec and *R*, can be found in Sections S4.5 and S4.6 of the Supporting Information.

31 $$\mathrm{TAC}=\left(a\times C_{p}+b\times \mathrm{heat}+c\times \mathrm{cool}+d\times \mathrm{elec}+e\times C_{\mathrm{memb}}\sum_{k}A_{\mathrm{memb}}^{k}\right)\times {\left(\frac{100}{R}\right)}^{f}$$

To obtain the multipliers we followed the guidelines from the U.S. Department of Energy for calculating marginal costs.^68^ Using the modified biorefinery design we developed in our previous work,^32^ we computed the change in TAC for a given perturbation in any of the components. Table S8 shows the obtained values of these multipliers and the corresponding units. When normalized, their values are consistent with unit energy costs reported in literature.^69^ While our approach to estimate *TAC* may appear more complex than other simpler methodologies, it captures the economic impact of the cascade within the biorefinery more accurately and remains computationally inexpensive. This provides a method that can yield more robust *TAC* estimates for the implementation of new processes within an established flowsheet.

## Results and Discussion

### Experimental Evaluation of Membrane Modules

Table 1 shows the range of operating conditions for the pervaporation experiments. In total seven experiments were conducted, and the goal was to evaluate the effects of two design variables (feed temperature and feed composition) on membrane performance. The range of values for these design variables was selected based on the anticipated operating conditions of the process design. We chose a range of low water concentrations since high BDO purity is desired on the final product of the cascade and because pervaporation is more effective when the feed is diluted in the main permeating component (water in this case). Furthermore, we selected a range of high temperatures near below the boiling point of the mixture, since these are conditions that would sustain a large driving force and keep the feed in the liquid phase throughout the process. Table 2 shows the chosen operating conditions and results of the experiments. To evaluate the effect of composition, experiments were performed at 363.15 K at varying BDO concentrations. To assess the effect of temperature, experiments were conducted at varying temperatures at 80 wt % BDO in the feed. Lastly, two additional points (experiments 2 and 4) were selected to help fill the design space, which is important for developing robust predictive models.

**Table 1:** Values for the Different Experimental Design Variables

design variable	values
permeate pressure, *P*_*p*_ (kPa)	1.08^a^
feed pressure, *P*_feed_ (kPa)	101.3
feed temperature, *T*_feed_ (*K*)	343.15–363.15
BDO content in the feed, *w*_feed,BDO_ (kg kg^-1^)	0.7–0.9

a Vacuum was applied using a Welch DryFast 2034 diaphragm pump. The system ran at the manufacturer’s specified maximum capacity of 29.6 inHg vacuum (1.08 kPa absolute pressure).

**Table 2:** BDO Rejection to the Retentate and Total Membrane Flux for Different Operating Conditions in Pervaporation Using Dense PVDF Hollow Fiber Membrane Module^a^

Exp. No	temperature (K)	BDO content in feed (kg kg^-1^) *w*_feed,BDO_	BDO content in permeate (kg kg^-1^) *w*_perm,BDO_	total flux (l m^–2^ h^–1^) *J*_LMH_	BDO rejection (%)
1a	353.15	0.80	0.032	0.19	96
1b	353.15	0.80	0.031	0.18	96
2	353.15	0.75	0.075	0.21	90
3a	358.15	0.85	0.119	0.19	86
3b	358.15	0.85	0.077	0.17	91
4	363.15	0.70	0.056	0.22	92
5a	363.15	0.80	0.112	0.25	84
5b	363.15	0.80	0.088	0.26	89
6	363.15	0.90	0.090	0.14	90
7	343.15	0.80	0.040	0.13	95

a Experiments with more than one entry represent replicates. Those without multiple row entries are single experiments.

Figure 3 shows the effects of feed composition and temperature on total flux, *J*_LMH_, and BDO rejection. As expected, for a constant feed composition of 80 wt % BDO (Figure 3a), the flux increases monotonically with temperature for the entire range due to the increased vapor pressure. For the experiments at 363.15 K (Figure 3b), although flux is expected to decrease with water content, due to the higher amount of competing BDO molecules, *J*_LMH_ drops very sharply at 90 wt % BDO in the feed. Similarly, the BDO rejection exhibits an inconsistent pattern with respect to temperature and feed composition, where a monotonic trend is typically expected.^70^ Rejection is highly sensitive to variations in permeate composition, and due to the low concentration of BDO in the permeate it can become more susceptible to measurement errors. This contributes to the observed inconsistencies and is also reflected in the relative size of the error bars of BDO rejection.

**Figure 3:**
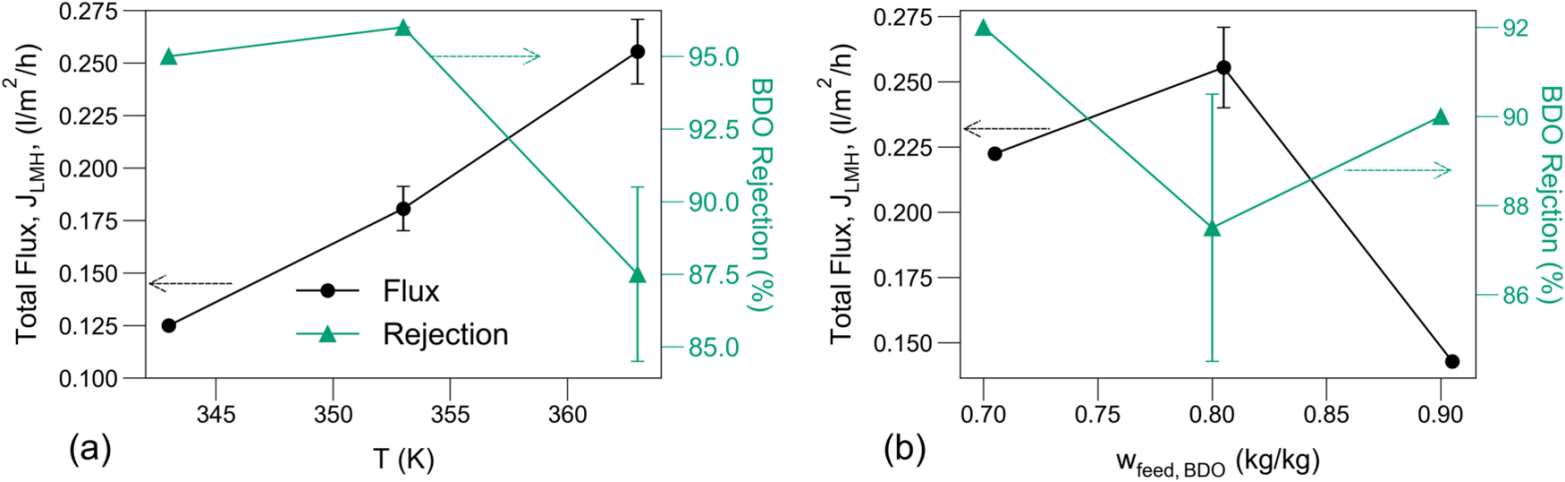
Total membrane flux (*J*_LMH_) and BDO rejection as a function of (a) feed temperature (*w*_feed,water_ = 0.2) and (b) water content in the feed (*T*_feed_ = 363 K) for the pervaporation of a BDO/water mixture. *P*_*p*_ = 1.08 kPa. Error bars represent the range of values for duplicate measurements, while symbols denote the average of each pair. Symbols without error bars correspond to single measurements and lines connecting points are used to guide the trend.

### Permeance Estimates

As is the case in most pervaporation studies, it is difficult to discern whether the inconsistent trends in the data stem entirely from measurement errors or are due to intrinsic membrane properties not captured by the model. As highlighted by Baker et al.,^71^ normalized terms such as permeance are less dependent on experimental conditions and offer a more reliable basis for evaluating pervaporation performance data than flux and rejection. We estimated BDO and water permeance and evaluated their relationship to feed temperature and composition as shown in Figure 4. For water, we now observe a more consistent trend with the design variables. The relationship between ln­(*Q*_water_) and (1/*T*) is a near-straight line, which is characteristic of an Arrhenius-type behavior and validates the use of the proposed permeance expression in eq 11. The positive slope of the regression line indicates a negative activation energy (*E*_A,water_ = −10.1 kJ mol^–1^), which, though less common, has previously been reported for water and alcohols on different types of pervaporation membranes.^72–76^ This suggests Δ*H*_sorp,water_ has a stronger influence on water pervaporation than *E*_diff,water_. When evaluating the influence of feed composition, it becomes evident that water permeance is nearly independent of water content. Therefore, *A*_conc,water_ was set to zero in the general permeance expression, eq 11, helping reduce the number of calibration parameters.

**Figure 4:**
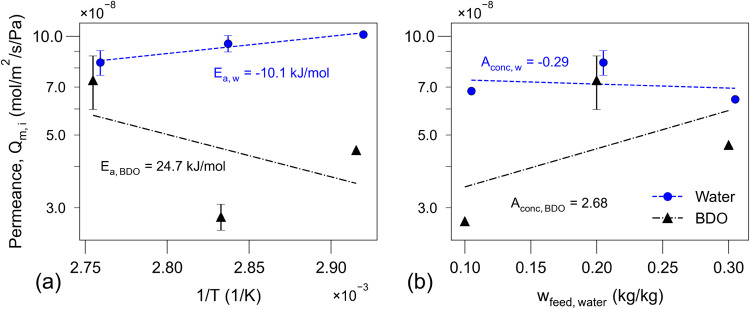
Arrhenius plot of the estimated molar permeances (*Q*_*m,i*_) of water (*Q*_*m*,water_) and BDO (*Q*_*m*,BDO_) as a function of (a) feed temperature (*w*_feed,water_ = 0.2 kg kg^–1^) and (b) water content in the feed (*T*_feed_ = 363 K). *P*_*p*_ = 1.08 kPa. Error bars represent the range of permeance values for duplicate measurements, while symbols denote the average of each pair. Symbols without error bars correspond to permeance estimates from single measurements. Error bars represent the range of permeance values for duplicate pairs, while points without error bars are permeance estimates from single experiments. The straight dashed lines represent the linear regression based on the corresponding linearized form of eq 11.

Similar to BDO rejection, for *Q*_BDO_ neither temperature nor composition displays a consistent pattern that can be captured by the proposed permeance model. Given that *Q*_water_ follows a stable pattern and is mostly influenced by flux, this suggests that the uncertainty can be primarily attributed to the measurement error of *w*_perm,BDO_. Changes in the permeance expression could help approach this challenge, however, model selection is considered outside of the scope of this study, where the goal is to assess uncertainty given the available data and set of expressions. To help simplify eq 11, we set *E*_A,BDO_ to zero and attribute all uncertainty of BDO permeance to *A*_conc,BDO_. Although it might be unsettling to derive empirical parameters from these inconsistent trends, it highlights the importance of the proposed Bayesian data-based methods. Under these conditions, treating **θ** as a random variable rather than a deterministic point estimate becomes a necessity for managing uncertainty and propagating it to the process design.

The reference temperature, *T*_ref_, is another variable whose value was fixed to reduce the number of parameters. *T*_ref_ has a small effect on the model behavior and is typically assigned an arbitrary value. We selected 353.15 K, the temperature of a control experiment with 100 wt % water in the feed (see Table S1), yielding the four calibration parameters shown in eq 32.

32 $$\theta ={\left[Q_{0,\mathrm{BDO}},A_{\mathrm{conc},\mathrm{BDO}},Q_{0,\mathrm{water}},E_{A,\mathrm{water}}\right]}^{T}$$

### Uncertainty Quantification

The MCMC was solved with the proposed MH algorithm. Figure 5 shows the resulting Markov chain evolution after 100,000 samples. The proposal width vector, *w*_*i*_, was tuned via inspection with the goal of ensuring an efficient exploration of the space of each parameter. Despite visible fluctuations, all chains successfully converged around a central value. This suggests an appropriate implementation of the algorithm and reliable sampling of the posterior density.

**Figure 5:**
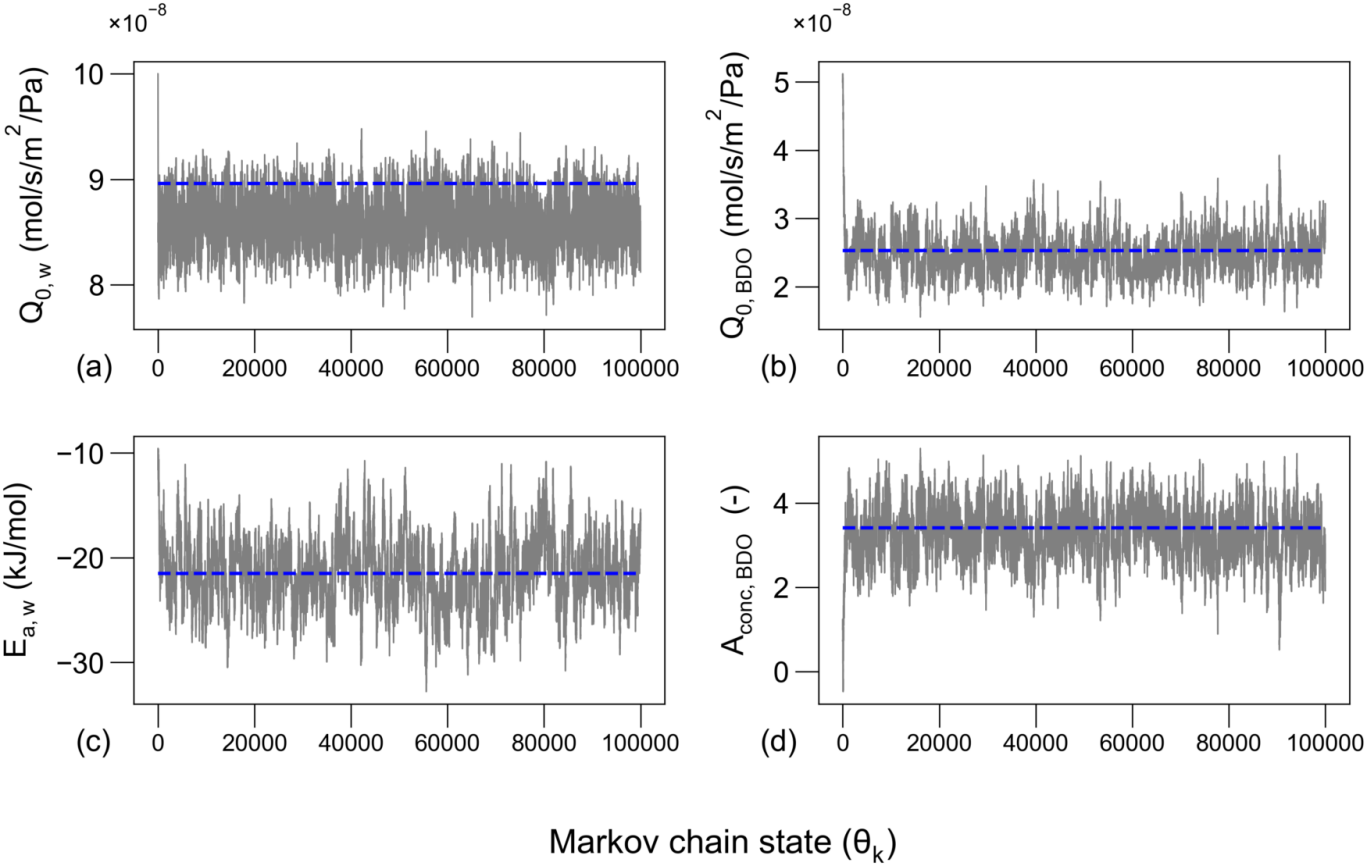
Evolution of the Markov chains of the permeance parameters, **θ** (eq 32), for 100,000 sample iterations. (a) Water pre-exponential permeance term, *Q*_0,*w*_, (b) BDO pre-exponential permeance term, *Q*_0,BDO_, (c) water permeance activation energy, *E*_a,*w*_, (d) BDO concentration empirical term, *A*_conc,BDO_. We assumed σ_LMH_ = 0.01 L m^–2^ h^–1^ and σ_perm,BDO_ = 0.0075 kg kg^–1^, which were determined experimentally. The entries of the proposal width vector, *w*_*i*_, can be found in Table S11 of the Supporting Information. The dashed horizontal lines represent the deterministic estimate of the parameters, θ_GLSR_.

The horizontal lines represent the deterministic estimates of ***θ*** obtained by minimizing the generalized least-squares regression (GLSR) loss function shown in eq 33. This was formulated as a nonlinear programming (NLP) optimization problem in Pyomo 6.5.^77^ and solved using IPOPT 3.12.13,^78^ an interior point solver. To prevent falling into local minima, the problem was repeatedly solved at different starting points sampled within carefully chosen bounds. More details of the initial guesses, bounds and the multistart approach can be found in Section S3.4 of the Supporting Information. ***Y*** is the experimental data, **∑** is the variance of measurement uncertainty and ***g*** (**θ**) is the predictor function, which is the solution to eqs 16–19 based on a given **θ**.

33 $$\theta_{\mathrm{GLSR}}=\underset{\theta }{\mathrm{argmin}}\left[{\left(g\left(\theta \right)-Y\right)}^{T}\Sigma^{-1}\left(g\left(\theta \right)-Y\right)\right]$$

***Y***, **∑** and ***g*** (**θ**) are the same as those used to solve the MCMC (see Section S.3.1 in the Supporting Information). Consequently, the mean values of the Markov chains, **θ̅**_MCMC_, closely align with the deterministic estimates, **θ**_GLSR_. The values of **θ̅**_MCMC_ and **θ**_GLSR_, however, are not always guaranteed to align even with an uninformative prior as was chosen here. Their agreement in this case suggests that fluctuations in the chain are not due to structural uncertainty (functional forms of the model that may lead to parameter identifiability issues) but instead are related to measurement uncertainty. The initial 5000 samples of the chain were discarded as burn-in, while the remaining 95,000 points were retained to represent the converged posterior density. Figure 6 shows the corner plot for the posterior density, *p*(**θ** *|**Y***). The histograms display symmetric, unimodal and well-defined bell shape marginal distributions for all parameters, which is a strong indicator of convergence and validates the underlying assumptions of normality. The remaining pairwise density plots reveal minimal correlation between BDO and water parameters, indicating **θ**_water_ and **θ**_BDO_ are interdependent and influenced by different subsets of the data. Water is the main permeating component and thus flux data is expected to have a stronger effect on **θ**_water_, while permeate composition data, and thus BDO rejection results, mostly affect **θ**_BDO_. As mentioned, BDO rejection exhibits a less consistent trend compared to flux, which explains the more elongated confidence ellipse between *Q*_0,BDO_ and *A*_conc,BDO_ compared to that between *Q*_0,water_ and *E*_a,water_. More informative priors could be employed to reduce the size of the confidence region. However, in this case it was found to have a small effect on the posterior. More details can be found in Figure S5 of the Supporting Information.

**Figure 6:**
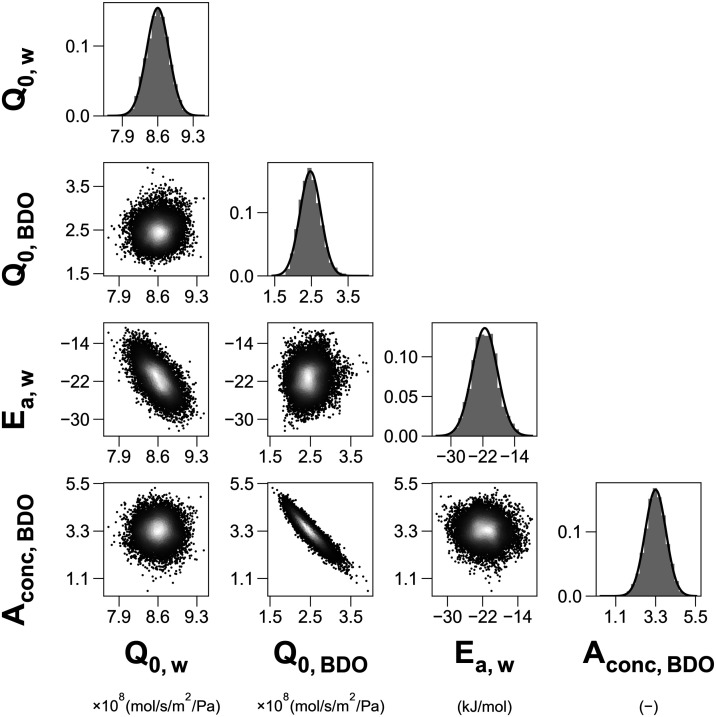
Corner pair plot showing the posterior density of the permeance parameters, ***p*** **(θ|** ***Y*** **)**, as estimated from the subset of converged samples in the MCMC solution. The off-diagonal plots show the correlation between individual parameters. The histograms on the diagonal entries show the distribution of each parameter, and their *y*-axes represent relative frequency. The solid curves represent fitted normal functions obtained from **θ**∼*N*(**θ̅**_MCMC_, Cov_MCMC_), where Cov_MCMC_ is variance-covariance matrix of **θ** computed from the MCMC results. The entries of Cov­(**θ**) used to develop this plot can be found in Table S12 of the Supporting Information.

Table 3 presents key statistics computed for this subset of converged samples, which includes the mean and 95% confidence intervals for the parameters and the comparison to the estimates from the GLSR frequentist method. We employed the approach outlined by Bates and Watts,^79^ full details of the calculation of CI are provided in Section S3.5. of the Supporting Information.

**Table 3:** Summary of the MCMC Solution and Comparison to the Deterministic Estimate Obtained from Generalized Least Squares Regression (**θ**_GLSR_)

permeance parameters (**θ**)	deterministic estimate (**θ**_GLSR_)	95% confidence intervals (CI_GLSR_)	mean value^c^ (**θ̅**_MCMC_)	95% confidence intervals^c^ (CI_MCMC_)
*Q*_0,water_ × 10^8^ (mol m^–2^ s^–1^ Pa^–1^)^a^	8.96	[7.55–10.37]	8.60	[8.17–9.03]
*Q*_0,BDO_ × 10^8^ (mol m^–2^ s^–1^ Pa^–1^)^b^	2.53	[0.65–4.41]	2.48	[1.97–3.09]
*E*_a,water_ (kJ mol^–1^)^a^	–21.5	[−41.1 to −1.92]	–21.5	[−27.7 to −14.9]
*A*_conc,BDO_ (−)^b^	3.41	[−0.33–7.16]	3.30	[2.14–4.41]

a Water permeance reduced to $Q_{\mathrm{water}}=Q_{0,\mathrm{water}}\times \exp\left(\frac{E_{a,\mathrm{water}}}{R}\left(\frac{1}{T_{\mathrm{ref}}}-\frac{1}{T_{\mathrm{feed}}}\right)\right)$ , *T*_ref_ = 353.15 K.

b BDO permeance reduced to *Q*_BDO_ = *Q*_0,BDO_ × exp (*A*_con,BDO_ × *w*_feed,water_).

c These statistics were computed from the converged subset of 95,000 samples in the MCMC result. The mean value is the average of the subset, while the confidence interval represents the 2.5% and 97.5% percentiles.

The value for the parameters reflect physically meaningful estimates and validate the proposed approach. *E*_A,water_ is comparable to the slope of the ln­(*Q*_water_) vs (1/*T*) plot, while *Q*_0,water_ is comparable to the estimate obtained from the control experiment. *Q*_0,BDO_ falls within the expected order of magnitude relative to *Q*_0,water_ and *A*_conc_ is within the range of values reported by previous researchers for pervaporation of water/alcohol mixtures.^2,11–13^

Together with the Markov chain evolution, these statistics can also be used to assess the level of uncertainty in the parameters. The pre-exponential permeance term for water, *Q*_0,water_, exhibits a notably narrower confidence interval and a smaller standard deviation relative to its mean compared to the corresponding term for BDO, *Q*_0,*BDO*_. This is also reflected in the shape of their Markov chains and reinforces that **θ**_water_ has a lower associated uncertainty compared to **θ**_BDO_. *E*_a,water_ and *A*_conc,BDO_ display the highest variability, appearing to capture most of the uncertainty observed in the data and reflecting the issues of identifiability for these parameters. Compared to the GLSR method, the CI values from MCMC are significantly narrower. This is expected given that GLSR is a frequentist approach that relies on a local curvature approximation and can overestimate uncertainty, especially when the data is small and noisy. This is more evident for less identifiable parameters (*Q*_0,BDO_, *E*_a,water_ and *A*_conc,BDO_). These results highlight the advantage of Bayesian inference approaches in producing refined parameter uncertainty estimates even when identifiability is challenging.

Despite the noticeable uncertainty in the permeance parameters, the model yields reliable predictions. Figure 7 shows parity plots comparing experimental data against model predictions using **θ** = **θ̅**_MCMC_. For flux, all predictions stay within 20% of the measured value, and the mean deviation is 9.0%. For BDO rejection, all predictions stay well within the 5% corridor, with the mean deviation being 2.5%. These levels of accuracy on the predictions of flux and permeate composition are comparable with those reported by Koch et al. in a similar combined experimental and modeling study.^13^

**Figure 7:**
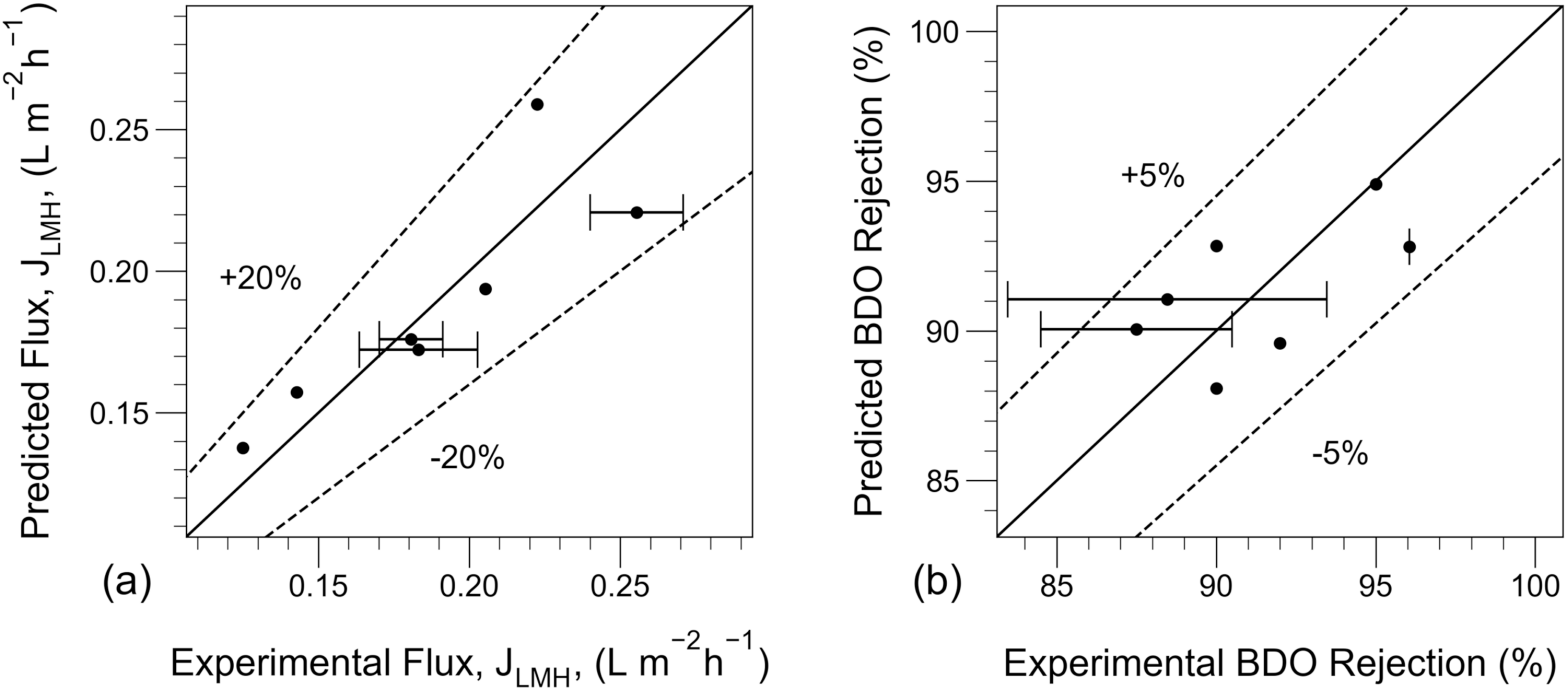
Parity plot comparing experimental and predicted values for (a) total membrane flux, *J*_LMH_, and (b) BDO rejection for the separation of BDO/water in a dense PVDF hollow fiber pervaporation membrane. *P*_*p*_ = 1.08 kPa. The solid line represents the 1:1 parity line, indicating a direct match between experimental and predicted values. Dashed lines define the deviation ranges from the 1:1 parity line, ±20% for flux and ±5% for BDO rejection. Error bars represent the range of values for duplicate measurements, while symbols denote the average of each pair. Symbols without error bars correspond to single measurements.

### Process Design and Uncertainty Propagation

For the proposed cascade, we assumed the feed is a binary BDO/water 26,980 kg/h stream with 70 wt % BDO. These conditions can reasonably be achieved by the BDO preconcentration SMB + distillation step. For the outlet stream we assumed 90 wt % BDO, which is an acceptable purity level for the upgrading of BDO. The process variables are listed in Table 4 along with their range of values. As mentioned, from all process variables, only feed temperature, *T*_feed_, and maximum allowable membrane area per stage, *A*_memb,max_, were varied, which are the typical conditions adjusted in pervaporation designs.^12,13^ Also, these variables are closely related to permeance uncertainty, with **θ**_water_ having a direct impact on flux, thereby influencing membrane area. Feed temperature is closely related to permeate composition and thus BDO recovery, which is also influenced by ***θ***_BDO_. As a simplification, *T*_inlet_, and *A*_memb,max_, are the same for all stages, which can be enforced by implementing eqs 34 and 35.

34 $$T_{\mathrm{inlet}}^{j}=T_{\mathrm{inlet}},\forall j\in \left\{1,2\cdot \cdot \cdot N\right\}$$

35 $$A_{\mathrm{memb},\max}^{j}=A_{\mathrm{memb},\max},\forall j\in \left\{1,2\cdot \cdot \cdot N\right\}$$

**Table 4:** List of the Design Variables (Decision, Fixed and Random) in the Membrane Cascade Process Design and Their Values

design variable	values
**Decision Variables**	
module *j* inlet temperature, *T*_inlet_^*j*^ (K)	[343.15–363.15]
module *j* maximum allowable area, *A*_memb,max_^*j*^ (m^2^)	[100–10,000]
**Fixed Variables**	
vacuum pressure, *P*_*p*_ (kPa)	7.0
inlet feed mass rate, (kg h^–1^)	27,587
inlet feedwater content, (wt %)	30
final streamwater content, (wt %)	10
**Random Variables**	
permeance parameters, **θ**	see Table 3

If we were to seek an optimal configuration of pervaporation with respect to the preconcentration step, the effect of other variables such inlet and outlet concentration could be further investigated. However, the main goal of this work is to evaluate the impact of membrane parameter uncertainty on process and economic metrics, and optimizing the design with respect to the overall biorefinery was considered outside the scope of the study.

To propagate uncertainty to the process design we randomly sampled 100 points from **θ**. Then, for each sample, given values of *T*_inlet_ and *A*_memb,max_ we solved the system of DAEs and developed the cascade design to compute key process performance metrics. Figure 8 shows the effect of *A*_memb,max_ on key process design metrics (total number of stages, total membrane area, total heat exchanger area and BDO recovery) and *TAC*. As shown in Figure 8c, decreasing *A*_memb,max_ leads to a rapid increase in the number of stages required to reach the target BDO concentration. Reducing the size of each membrane leads to a lower stage cut, which reduces the temperature drop per module and results in more frequent reheating of the mixture. Maintaining a high temperature throughout the process keeps a strong driving force and high flux at each stage and requires less total membrane area to achieve the desire separation, as shown in Figure 8a. In contrast, at large *A*_memb,max_ values, lower driving forces emerge, leading to suboptimal fluxes and requiring more membrane area. However, sustaining a large temperature does not benefit all process metrics as it requires a higher heat exchanger area, causes increased BDO losses to the permeate and slightly reduces its recovery. Lower values of *A*_memb,max_ are still preferred since BDO recovery remains close to 100% and membrane unit costs are substantially higher than those of heat exchangers. This is reflected in Figure 8e, where lower values for TAC are obtained as *A*_memb,max_ decreases.

**Figure 8:**
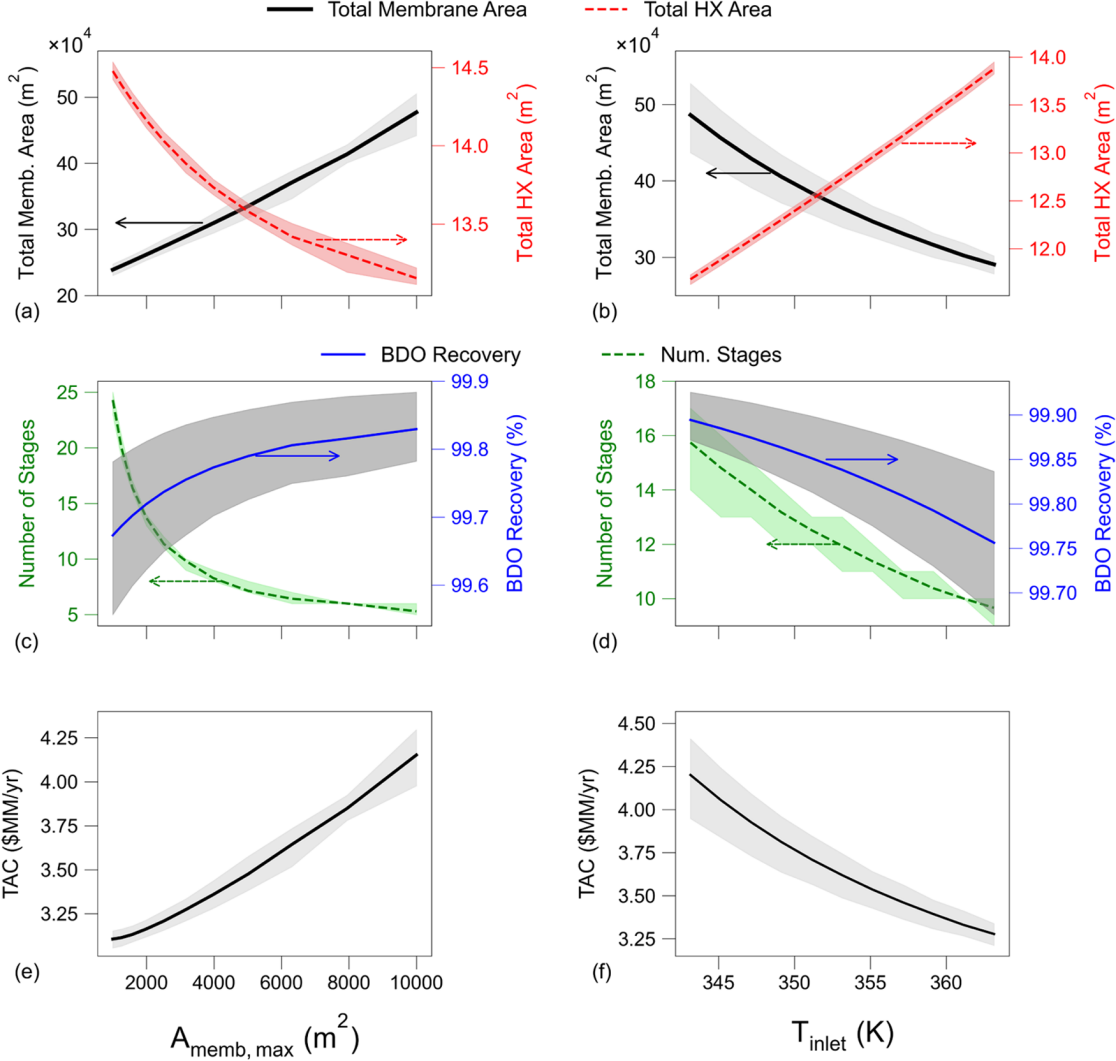
Effect of maximum allowable membrane area per stage, *A*_memb,max_, (left) and feed inlet temperature, *T*_inlet_, (right) on the performance of the membrane cascade design for a BDO/water separation. Metrics of the cascade design: (a, b) Total membrane area and heat exchanger area, (c, d) BDO recovery and number of required stages, and (f) Total annual cost (TAC). Solid and dashed lines represent mean values of each metric, while the shaded areas show 95% confidence intervals, computed from the uncertainty propagation analysis. (a, c, e) were evaluated at *T*_inlet_ = 363.15 K and (b, d, f) were evaluated at *A*_memb,max_ = 3200 m^2^.

Despite the benefits of increasing the number of stages, this raises the number of unit operations, which can make the process design challenging to implement. In practice, there is an upper limit to the number of pervaporation stages allowed. This is a constraint highlighted by the work from previous researchers that have designed pervaporation cascade systems, with Constantino et al. reporting 12 stages,^3^ Vatankhah et al.^52^ reporting 10, Tan et al.^23^ 11 and Zhang et al.^67^ reporting a maximum of 7 stages. From our analysis, *A*_memb,max_ = 3200 m^2^ would on average yield 10 pervaporation stages. At this value of *A*_memb,max_ we then explored the effect of *T*_inlet_ on the process. A similar trade-off between total membrane and heat exchanger area is observed in Figure 8b, where increasing the feed temperature helps sustain a large flux and lower membrane area, but at the expense of a large heat exchanger duty and area and slightly reduced BDO recovery. However, as previously determined, recovery remains close to 100% and thus its effect is minimal. A high inlet temperature should then be preferred to minimize TAC.

Following the process evaluation, we now examine the uncertainty propagation results to better understand how each parameter influences specific portions of the design. The findings obtained from the MCMC solution indicate that θ_water_ and θ_BDO_ are interdependent and thus influenced by different subsets of the data. **θ**_water_ is primarily associated with flux and has a stronger impact on membrane, while **θ**_BDO_ is related with permeate composition and primarily impacts recovery. As indicated by the MCMC results, the associated uncertainty of **θ**_water_ was considerably lower than that of **θ**_BDO_. This is reflected in the lower variability propagated to the total calculated membrane area compared to BDO recovery, as measured by the sizes of the confidence intervals relative to their nominal values (shaded areas in Figure 8).

### Pervaporation Process Design

Based on the previous economic assessment we selected the operating conditions to develop the final cascade design. As mentioned, *A*_memb,max_ was fixed at 3200 m^2^, which on average would yield 10 stages. Table S12 shows detailed dimensions of each membrane module (area per submodule, submodule diameter and fiber length). To determine *T*_inlet_ we selected a temperature for the mixture to remain in the liquid phase. The upper bound of *T*_inlet_ is the bubble point of the feed at the inlet pressure, with a safety margin of ∼10 K below this point to prevent any risk of vaporization. For *P*_feed_ = 101 kPa at the given compositions, this corresponds to a temperature range of approximately 363–370 K. Although increasing pressure would permit higher temperatures, this would require extrapolation outside the tested experimental conditions, adding complexity in the uncertainty quantification and extending beyond the focus of this work. *T*_inlet_ = 363.15 K was selected given this was the highest temperature evaluated. Table 5 shows the details of the optimal process design. To determine uncertainty of our design calculations we randomly drew 100 samples from ***p*** (***θ*** | ***Y***). *C*_*p*,modules_ dominates the total purchase equipment cost, accounting for over 70% of *C*_*p*_, and when considering yearly replacement cost, the membrane modules account for 45% of TAC. This emphasizes the importance of minimizing membrane area, despite additional LPS costs or reduced BDO recovery. Finally, it is also important to note that the confidence interval of the reported TAC is narrow relative to its nominal value. This is attributed to the narrow confidence interval of total membrane area, the main component of TAC. Even though experimental data exhibited significant uncertainty and the estimate of BDO recovery had a wide confidence interval, TAC remained marginally affected, an insight that would not have been achieved without our proposed approach.

**Table 5:** Details of the Final Process Design Recommendation^a^

metric	estimated value
number of stages	10^b^
BDO recovery (%)	99.75 ± 0.10
total membrane area, ∑*A*_memb_^*k*^ (m^2^)	28,060 ± 1,080
total heat exchanger area, (m^2^)	13.9 ± 0.06
**Utilities**	
total low-pressure steam (LPS) duty (MW)	4.07 ± 0.01
total cooling duty, chilled water (CHW) (MW)	9.61 ± 0.03
total electricity duty (kW)	10.22 ± 0.32
OPEX ($MM/yr)	2.07 ± 0.02
**Purchase Equipment Costs (** ***C***_***p***_ **)**	
condenser, *C*_*p*,cond_ ($MM)	0.34 ± 0.0004
vacuum pump, *C*_*p*,vac‑pump_ ($MM)	0.06 ± 0.0001
preheaters, ∑*C*_*p*,preheater_^*k*^ ($MM)	0.79 ± 0.011
feed pump, ∑*C*_*p*,feed‑pump_^k^ ($MM)	0.05 ± 0.002
membrane modules, ∑*C*_*p*,modules_^*k*^ ($MM)	2.91 ± 0.11
CAPEX ($MM)	4.14 ± 0.14
**Final Economic Estimate**	
total annualized cost (TAC) ($MM/yr)	3.27 ± 0.05

a The reported values are mean estimates derived from 100 randomly drawn samples, with the uncertainties (±) representing two standard deviations from the mean. *T*_inlet_ = 363.15 K and *A*_memb,max_ = 3200 m^2^. For details regarding the mass and energy balances of each stage please refer to Table S14 of the Supporting Information.

b From the 100 random designs, 10 membrane stages were estimated in 73% of the designs and 9 stages were estimated in 27% of the designs.

### Comparison to Simple Distillation

As mentioned, the 70% BDO feed comes from an SMB + distillation scheme. However, this stream can be further purified to 90% by increasing the capacity of the distillation column and adjusting its operation. This leads to an increase in reboiler duty of 4.52 MW, which is comparable to the 4.07 MW duty for pervaporation reported in Table 5. This close alignment is expected given the high relative volatility ratio between water and BDO and thus low reflux ratio achieved in the distillation. The larger distillation column also adds $0.2 MM in CAPEX, which is far smaller than 4.14 $MM of pervaporation, and leads to a TAC of 0.43 $MM/yr.

Despite this economic disadvantage, the goal of this work was not to minimize costs for BDO recovery. The aim was to develop and apply a framework that integrates membrane fabrication, experimental data, and Bayesian inference to quantify and propagate uncertainty throughout process design to understand its effect in high-level metrics. Using pervaporation allowed us to demonstrate this methodology for a case study relevant to industry. It also helped highlight the benefits of Bayesian statistics over deterministic/frequentist approaches, particularly when parameter identifiability is challenging due to limited and uncertain data.

## Conclusions

We have comprehensively evaluated pervaporation processes for purifying 2,3-butanediol (BDO) from a preconcentrated (70 wt %) stream to high purity (>90 wt %), via PVDF-coated hollow fiber membranes developed in this work. Our approach included: (1) selecting experimental conditions relevant to the anticipated process design, (2) experimentally testing the membrane performance (flux and BDO rejection) under these conditions, (3) estimating membrane parameters and quantifying their uncertainty via Bayesian inference, (4) developing a pervaporation-based process design with uncertainty propagation, and (5) providing a final design recommendation and economic evaluation. Dense PVDF coated membranes were fabricated with high water/BDO separation factors (37–120). Modules were assembled and tested at concentration and temperature ranges favorable for a pervaporation process design. Experimental results showed high membrane flux (>0.2 LMH) and >95% BDO rejection can be achieved by these membranes, which is a significant advance of this work. However, uncertainty in data and inconsistent patterns in estimated permeance motivated the use of Bayesian statistics to infer the posterior density distribution of the parameters via Monte Carlo Markov Chain (MCMC) methods. The posterior obtained through this method yielded significantly more narrow confidence intervals compared to a frequentist approach.

A multistage cascade pervaporation system was designed, and uncertainty in the parameters was propagated. This study reveals that the uncertainty mostly impacted BDO recovery (leading to a broad confidence interval in this metric), while the total membrane area estimate remained narrow. Given the challenge of limited data and our proposed simplified permeance model, achieving reliable and low uncertainty estimates for total membrane area is also a significant outcome of this work. Since BDO recovery remained ∼100%, it had minimal effect on the overall process and the narrow confidence interval of total membrane area ensured low uncertainty in total annualized cost (TAC) calculations as well. Without the present uncertainty quantification approach, it would have been challenging to achieve these insights. Treating parameters as random variables enabled robust uncertainty propagation, leading to a confident and reliable final process design recommendation. Although this study highlighted the potential of Bayesian uncertainty quantification for pervaporation membranes and evaluation under uncertainty, further exploration is also needed. Design and optimization under uncertainty would achieve better integration of pervaporation with other separation technologies in biorefineries and enable a more comprehensive techno-economic analysis that can be compared to alternative state-of-the-art BDO recovery process designs. This approach could also support the optimal recovery of other platform chemicals through membrane-based processes beyond pervaporation.

## Supplementary Material





## Data Availability

The data underlying this study are available in the published article and its Supporting Information.

## Notes

Additional details for the membrane mechanism, fabrication and testing; SEM images of the coated PDVF fibers; photograph of the experimental setup; physical and thermodynamic properties of the system; mathematical models; assumptions of the techno-economic assessment; algorithms to solve the system of differential equations and to perform the Bayesian uncertainty quantification; comparison against deterministic/frequentist approaches; effect of the prior distribution; sensitivity analysis of the final BDO purity; development of correlations and surrogate expressions; detailed Markov chain and Monte Carlo results; complete mass and energy balance and detailed dimensions of the final process design recommendation (PDF)
Raw data from Aspen simulations to develop the surrogate expressions (XLSX)
