# Supplementary material for: Pervaporative Dehydration of 2,3-Butanediol by Dense Poly(vinylidene fluoride) Hollow Fiber Membranes: Parameter Estimation, Process Design, and Technoeconomic Evaluation under Uncertainty
Source: Ind Eng Chem Res. 2025 Aug 12;64(34):16770–85. doi: 10.1021/acs.iecr.5c01198 (PMC12395413; doi:10.1021/acs.iecr.5c01198)
Supplement: Supplementary file 1 [file ie5c01198_si_001.pdf]

Supporting Information

for

Pervaporative Dehydration of 2,3-butanediol by Dense Polyvinylidene Fluoride Hollow Fiber Membranes: Parameter Estimation, Process Design, and Technoeconomic Evaluation under Uncertainty

Marco Avendano [a §], Blake Trusty [b§], Shailesh Dangwal [b], Zachary Coin [b], Syed Islam [b], Sankar Nair [a], Ramesh Bhave [b*], and Matthew J. Realff [a*]

[a] *School of Chemical and Biomolecular Engineering, Georgia Institute of Technology, Atlanta, GA, 30332, USA*

[b] *Oak Ridge National Laboratory, Chemical Sciences Division, Oak Ridge, TN, 37830, USA*

[§]M.A. and B.T. contributed equally to this work

[*]Co-corresponding authors

To whom all correspondence should be addressed.

[*] E-mail: bhaverr@ornl.gov

[*] E-mail: matthew.realff@chbe.gatech.edu

Number of pages: 55

Number of supporting tables: 14

Number of supporting figures: 8

Supporting Information Content

S1. Membrane fabrication and testing 4

S1.1 Fabrication of a dense, defect free PVDF coating layer on porous PVDF fibers. 4

S1.2 Membrane testing: control experiment 4

S1.3 Optimization of coating preparation 5

S2. Physical and Thermodynamic Properties 6

S2.1 UNIQUAC thermodynamic model 6

S2.2 Enthalpy of vaporization, constant pressure heat capacity and vapor pressure 6

S2.3 Pure component physical properties 7

S3. Uncertainty Quantification 9

S3.1 Bayesian Framework 9

S3.2 Bayesian inference via Markov chain Monte Carlo sampling (MCMC) 11

S3.3 Effect of Prior Distribution 13

S3.4 Bounds on Variables and Multi-start Solver for the GLSR Method 14

S3.5 Frequentist Uncertainty Quantification 15

S4. Mathematical Models 16

S4.1 Derivation of mass and energy balances 17

S4.2 Solution to the system of differential algebraic equations 19

S4.3 Derivation of the quadratic equation to solve for $yBDO$, $Jwater$ and JM 20

S4.4 Economic model – equipment sizing 22

S4.5 Economic model – equipment costing, purchase equipment cost ($) 28

S4.6 Economic model – utilities 29

S4.7 BDO recovery 30

S4.8 Chemical engineering cost index 30

S5. Correlations/Surrogates 31

S5.1 Vacuum system correlations 31

S5.2 Total annualized cost correlation 32
S6. Markov chain Monte Carlo hyperparameters and results 36
S7. Process design recommendation 37
S7.1 Mass and energy balance results 37
S7.2 Sensitivity analysis 37
S8. Supplemental Tables 38
S9. Supplemental Figures 48

S1. Membrane fabrication and testing

S1.1 Fabrication of a dense, defect free PVDF coating layer on porous PVDF fibers.

Figure S2 shows the SEM images of coated PVDF fibers with different fabrication conditions. **Figure S2 a, d, g, and j** show the SEM image of porous pretreated fiber. Due to the charging issue, higher resolution images could not be obtained. After coating a clear distinction with the pretreated fiber was observed. **Figure S2b** and **Figure S2c** show fibers coated with different dipping time of 30s and 120s. The coating layer was again thick and fragmented, which was attributed to either long dipping times or high amounts of crosslinker. Different amounts of crosslinker Easaqua was used in the next coating procedure, the results of which can be seen in **Figure S2e** and **Figure S2f**. However, this still resulted in thick and broken coatings.

Moving forward, the dipping time was reduced to 10s and PVDF dispersion concentration was diluted in water by a factor of 2, while varying the amount of crosslinker. **Figure S2h** and **Figure S2i** show SEM images with 0.4g of Easaqua and 0.2g of Easaqua, respectively. **Figure S2h** and **Figure S2i** still showed clear cracks but with much improved PVDF coated layer quality. This shows shorter dipping time, and lower PVDF dispersion concentration was helpful in improving the coating quality. Lastly, with 50% diluted PVDF dispersion solution, we investigated lower dipping time of 5s and 10s, which did help in marginally improving the coating quality. From these results it was concluded that shorter dipping times, lower PVDF dispersion solution concentration and lower crosslinker amount was beneficial in improving the coating quality. Final optimized coating conditions indicated in the main text resulted in a coating layer able to reject >95% of BDO while still maintaining a high cross membrane water flux.

S1.2 Membrane testing: control experiment

The membrane was tested using the setup in **Figure S3**. A control experiment with a pure water feed was conducted to test the permeance capability of the membrane, the results are shown in **Table S1.** Water permeance, $Q_{m,water}$, can be computed using equations 2-9 from the main text and requires only knowing density $\left(\rho_{water} = 53.78 \frac{mol}{L}\right)$ and vapor pressure $(P_{sat,wat} = 47{,}420\ Pa)$ at 353 K. This

gives $Q_{m,water} = 9.67x10^{-8}\ mol\,m^{-2}\ s^{-1}\ Pa^{-1}$, which is comparable to the value of the pre-exponential factor $Q_{0,water} = 8.04x10^{-8}\ mol\,m^{-2}\ s^{-1}\ Pa^{-1}$ obtained in Table 3 of the main text.

S1.3 Optimization of coating preparation

All measurements reported in the main text of this manuscript were done with the same batch of coated PVDF membranes. However, data that was initially deemed outside the scope of this work looked to optimize the number of coatings to achieve the desired membrane performance.

Figure S4 and **Table S2** show the effect of the number of coating layers. These data are from experiments with different batches of prepared membranes and show that the high BDO rejection rates (96%) and permeate flux (0.19-0.25 $L^{1}m^{-2}h^{-1}$) can be reproduced with 4 coating layers, which is the preparation method for the membrane materials reported in Table 2 of the main manuscript and beyond.

S2. Physical and Thermodynamic Properties

S2.1 UNIQUAC thermodynamic model

The UNIQUAC thermodynamic model was chosen to estimate the activity coefficients, γ_i, for each of the component i at varying temperatures, T(K), and molar compositions, x_i, of BDO/water mixtures. Equations (S1) – (S8) show the utilized expressions.

$$\phi_i = \frac{r_i x_i}{\sum_k r_i x_k} \tag{S1}$$

$$\theta_i = \frac{q_i x_i}{\sum_k q_k x_k} \tag{S2}$$

$$I_i = \frac{z}{2}(r_i - q_i) + 1 - r_i \tag{S3}$$

$$\tau_{j,i} = exp\left(\frac{a_{ij}}{RT}\right) \tag{S4}$$

$$t_i = \sum_k \theta_i \tau_{k,i} \tag{S5}$$

$$ln\left(\gamma_i^C\right) = ln\left(\frac{\phi_i}{x_i}\right) + \frac{z}{2} q_i\, ln\left(\frac{\theta_i}{\phi_i}\right) + I_i - \frac{\phi_{s,i}}{x_i}\sum_k x_k I_k \tag{S6}$$

$$ln\left(\gamma_i^R\right) = q_i\left[1 - ln(t_i) - \sum_k \frac{\theta_{s,l}\tau_{i,k}}{t_k}\right] \tag{S7}$$

$$ln(\gamma_i) = ln\left(\gamma_i^C\right) + ln\left(\gamma_i^R\right) \tag{S8}$$

Table S3 lists the values of the relative Van der Waals volumes, r_i, and surface areas, q_i, for BDO and water. **Table S4** presents the binary interaction parameters, a_{ij}, which as mentioned were fitted to BDO/water vapor-liquid equilibrium (VLE) data at 10 kPa by the authors DECHEMA series. Following standard assumption, self-interaction parameters were set to zero ($a_{ii} = 0$). Lastly, R is the universal gas constant, which set to 1.987 $\frac{cal}{mol\,K}$, and z is the coordination number, which was set to 10.

S2.2 Enthalpy of vaporization, constant pressure heat capacity and vapor pressure

Table S5 lists the enthalpy of vaporization and liquid constant-pressure heat capacity values for BDO and water, referenced at 40°C. These values were obtained from the single component database in

Aspen Plus[1]. The reference temperature was chosen because the permeate is primarily composed of water (>99 wt.%), and 40°C approximates its dew point at the fixed vacuum pressure of 7 kPa. While temperature corrections are typically necessary, the effect on liquid mixtures is negligible and these properties were treated as constants, as detailed in the energy balance in the main text.

Table S6 shows the Antoine constants used to estimate vapor pressure, *P* (bar), as a function of temperature, *T* (K). Constants were obtained from the NIST Chemical Webbook Database[2].

S2.3 Pure component physical properties

The molar density of a pure component i as a function of temperature, $\rho_i(T)$, is determined with the following expression, where $\rho_i^0 \left(\frac{kmol}{m^3}\right)$ and T^0 (K) are the reference molar density and temperature, respectively. $\beta_i \left(\frac{1}{K}\right)$ is the cubic expansion coefficient and is fitted for each component.

$$\rho_i(T) = \rho_i^0 \exp\left[-\beta_i(T - T^0)\right] \tag{S9}$$

Table S7 shows the value of the fitted coefficients. The data used to fit the coefficients was obtained from Aspen Plus simulations and can be found in the Supplemental Data. The temperature range and accuracy of the correlations can also be found in the Supplemental Data.

The molar density of the mixture, $\rho(T) \left(\frac{kmol}{m^3}\right)$, was obtained using a Redlich-Kister geometric expansion for non-ideal binary mixtures[3], equation (S10)). The density of an ideal mixture is $\rho_{ideal}(T)$ $\left(\frac{kmol}{m^3}\right)$, x_i is molar composition and A_n are the Redlich-Kister coefficients.

$$\rho(T) = \rho_{ideal}(T) + x_{BDO}(1 - x_{BDO}) \sum_{n=0}^{N} A_n (2x_{BDO} - 1)^n \tag{S10}$$

$$\frac{1}{\rho_{ideal}(T)} = \frac{1}{\sum \frac{1}{\rho_i(T)}} \tag{S11}$$

The geometric expansion was truncated to $N = 1$, giving $A_0 = 2.90 \frac{kmol}{m^3}$ and $A_1 = -3.03 \frac{kmol}{m^3}$. These were also obtained by fitting the expressions to Aspen Plus simulation data that can be found in the

Supplemental Data. The correlation predicts molar density with a root mean square error (RMSE) value $O(10^{-2})$. The data spans temperature and molar composition ranges present the experiments and anticipated in the proposed process design. Details of the quality of fit and range of the correlation can be found in the Supplemental Data.

Lastly, we also obtained an expression for viscosity of the mixture as function of temperature and feed molar composition, $\mu(T,x_{BDO})\left(\frac{kg}{m \cdot s}\right)$,using a variation of the Andrade's equation[4]. Here, the coefficients to fit are reference viscosity, $\mu^0\left(\frac{kg}{m \cdot s}\right)$ and temperature T^0 (K), values and exponential molar composition-based, A, and temperature-based, B (K), empirical constants.

$$\mu(T) = \mu^0 \exp\left(Ax_{BDO} + \frac{B}{T - T^0}\right) \tag{S12}$$

Table S8 shows the fitted coefficients. The data was obtained from Aspen Plus simulations and the proposed expression fits it exceptionally well. Details can be found in the Supplemental Data.

S3. Uncertainty Quantification

S3.1 Bayesian Framework

The parameter estimation is performed by identifying the set of values that minimize error between model predictions and experimental measurements. However, this estimation is subject to aleatoric (instrument error) and epistemic (model) uncertainty, the latter arising from the semi-empirical nature of the permeance expressions. As the uncertainty carries over to the process, deterministic estimates of $\boldsymbol{\theta}$ may fail capture the variability and lead to unreliable results. It is therefore important to properly quantify uncertainty and identify the most parameters most sensible to this uncertainty.

Thermodynamic terms (γ_i and $P_{sat,i}$) were derived from established methods and references and thus can be treated as deterministic variables in the model. The uncertainty can then be attributed entirely to $\boldsymbol{\theta}$ and $\boldsymbol{Q_m}$. These are now treated as random variables in the model and thus propagate the uncertainty of the experimental data to the process design and economic analysis. Bayesian statistics provides a systematic approach for addressing this challenge and inferring the unknown joint distribution of $\boldsymbol{\theta}$ given observed information, $\boldsymbol{Y}$, (the measured data). This unknown distribution is called the posterior density, $p(\boldsymbol{\theta}|\boldsymbol{Y})$ and can be calculated through the Bayes theorem[5].

$$p(\boldsymbol{\theta}\,|\,\boldsymbol{Y}) = \frac{p(\boldsymbol{Y}\,|\,\boldsymbol{\theta})p(\boldsymbol{\theta})}{p(\boldsymbol{Y})} \tag{S13}$$

Here, the experimental data consists of m observations (experiments) each with n_Y measurement responses, $\boldsymbol{Y} \in \mathbb{R}^{\boldsymbol{n_Y}\times\boldsymbol{m}}$, and n_X design variables, $\boldsymbol{X} \in \mathbb{R}^{\boldsymbol{n_X}\times\boldsymbol{m}}$. In this study we consider two measurement responses, ($n_Y = 2$), which are the total membrane flux, J_{LMH} ($Lm^{-2}h^{-1}$), and weight fraction of BDO in the permeate, $w_{permeate,BDO}$ ($kg\ kg^{-1}$). Likewise, there are two design variables, ($n_X = 2$), which are feed composition, $w_{feed,water}$ ($kg\ kg^{-1}$), and temperature, T.

$$\boldsymbol{Y} = \left\{\left(J_{LMH,i}, w_{permeate,BDO,i}\right)\right\}_{i=1}^{m} \tag{S14}$$

$$\boldsymbol{X} = \left\{\left(w_{feed,water,i}, T_i\right)\right\}_{i=1}^{m} \tag{S15}$$

Next, we assume that a predictor function, $\boldsymbol{g}(\boldsymbol{\theta})$, can be used to estimate $\boldsymbol{Y}$ given $\boldsymbol{X}$. Here, $\boldsymbol{g}:\mathbb{R}^{\boldsymbol{n_X}\times \boldsymbol{M}}\rightarrow \mathbb{R}^{\boldsymbol{n_Y}\times \boldsymbol{M}}$, represents an operator returning the solution (J_{LMH} and $w_{perm,BDO}$) to the following system of non-linear equations (this is the solution-diffusion model that's outlined in equations 2-14 of the main text, please refer to the manuscript for a detailed explanation).

$$J_{M,i} = Q_{m,i} \times DF_i \tag{S16}$$

$$Q_{m,i} = f\left(w_{feed,water}, T_{feed}; \theta_i\right) = Q_0 \times \exp\left(A_{con} \times w_{feed,water} + \frac{E_A}{R} \times \left(\frac{1}{T_{ref}} - \frac{1}{T_{feed}}\right)\right) \tag{S17}$$

$$DF_i = \hat{f}^{G,hypoth}_{feed,i} - \hat{f}^{G}_{perm,i} = \hat{f}^{L}_{feed,i} - \hat{f}^{G}_{perm,i} \tag{S18}$$

$$\hat{f}^{L}_{feed,i} = x_i \gamma_i P_{sat,i} \Lambda_i \tag{S19}$$

$$\hat{f}^{G}_{perm,i} = y_i \phi_i P_p \tag{S20}$$

$$\sum x_i = 1 \tag{S21}$$

$$\sum y_i = 1 \tag{S22}$$

$$J_{M,i} = y_i J_M \tag{S23}$$

Thus, given values of $w_{feed,water}$ and T, J_M $(mol\, m^{-2}\, s^{-1})$ and y_{BDO} $(mol\, mol^{-1})$ can be obtained. These are mole-based quantities and were converted to the appropriate units of $\boldsymbol{Y}$. Lastly, we account for the effect of uncertainty error ε in the data using the following expression. Assuming ε is normally distributed, with mean zero and covariance $\boldsymbol{\Sigma}$, it follows $\boldsymbol{Y}$ is also normally distributed.

$$\boldsymbol{Y} = \boldsymbol{g}(\boldsymbol{\theta}) + \boldsymbol{\varepsilon} \tag{S24}$$

$$\varepsilon \sim N(\boldsymbol{0}, \boldsymbol{\Sigma}) \tag{S25}$$

The likelihood function, $p(\boldsymbol{Y} \mid \boldsymbol{\theta})$, can then be explicitly represented by the following equations. We assumed $\boldsymbol{\Sigma}$ is a diagonal matrix with the variance of uncertainty of each measurement, σ_i^2, as the diagonal elements. Also, σ_i^2 is assumed to be constant across $\boldsymbol{X}$ (homoscedasticity) and is determined from replicates for each measurement i [6].

$$p(\boldsymbol{Y}\,|\,\boldsymbol{\theta}) = \frac{1}{(2\pi)^{\frac{n_Y}{2}}|\boldsymbol{\Sigma}|^{\frac{1}{2}}}\exp\left[-\frac{1}{2}\boldsymbol{h}(\boldsymbol{X},\boldsymbol{Y},\boldsymbol{\theta})\right] \tag{S26}$$

$$\boldsymbol{h}(\boldsymbol{Y},\boldsymbol{\theta}) = (\boldsymbol{g}(\boldsymbol{\theta}) - \boldsymbol{Y})^T\boldsymbol{\Sigma}^{-1}(\boldsymbol{g}(\boldsymbol{\theta}) - \boldsymbol{Y}) \tag{S27}$$

$$\boldsymbol{\Sigma} = \begin{bmatrix} \sigma_1^2 & \cdots & 0 \\ \vdots & \ddots & \vdots \\ 0 & \cdots & \sigma_{n_Y}^2 \end{bmatrix} \tag{S28}$$

For parameter estimation problems, the argument of the likelihood, $\boldsymbol{h}(\boldsymbol{Y},\boldsymbol{\theta})$, is known as the weighted residual, or fitness term. Given $\boldsymbol{X}$ and $\boldsymbol{Y}$, the likelihood becomes only a function of $\boldsymbol{\theta}$ and increases as the fitness term approaches zero and measures how close the model can predict a given set of observations[5]. Lastly, $p(\boldsymbol{\theta})$ is the prior density of $\boldsymbol{\theta}$ before any information is presented and $p(\boldsymbol{Y})$ is the marginal distribution of $\boldsymbol{Y}$, also known as the evidence.

$$p(\boldsymbol{Y}) = \int p(\boldsymbol{Y}\,|\,\boldsymbol{\theta})p(\boldsymbol{\theta})d\boldsymbol{\theta} \tag{S29}$$

In practice, this expression is not possible to compute and is often disregarded since is not directly a function of $\boldsymbol{\theta}$ and only serves as a normalization factor. This leads to the following simplification, where only the likelihood and the prior density functions are needed to infer the posterior density.

$$p(\boldsymbol{\theta}\,|\,\boldsymbol{Y}) \propto p(\boldsymbol{Y}\,|\,\boldsymbol{\theta})p(\boldsymbol{\theta}) \tag{S30}$$

S3.2 Bayesian inference via Markov chain Monte Carlo sampling (MCMC)

Estimating the likelihood and prior functions over the entire parameter space is often computationally infeasible. Instead, researchers employ stochastic sampling techniques, with Markov chain Monte Carlo (MCMC) methods being a popular choice. In these methods, the invariant distribution of the chains is also the posterior density, meaning that convergence implies successful inference of the underlying parameter distribution. The Metropolis-Hastings (MH) algorithm is one of the most widely used implementations of MCMC. The following are the steps of the proposed algorithm based on the guidelines defined by previous studies[7-12]:

1. $k \rightarrow 0$. Initialize the chain with $\boldsymbol{\theta}_k = \boldsymbol{\theta}_0$, where $\boldsymbol{\theta}_0$ is the initial state of $\boldsymbol{\theta}$ and can be interpreted as an initial guess.

2. $k \rightarrow k+1$. Propose a new candidate state, $\boldsymbol{\theta}^*$, based on the current state, $\boldsymbol{\theta}_k$. This is done by sampling from a proposal distribution $q(\boldsymbol{\theta}^* \mid \boldsymbol{\theta}_k)$. We chose a multivariate normal distribution for q.

$$\boldsymbol{\theta}^* \sim q(\boldsymbol{\theta}^* \mid \boldsymbol{\theta}_k) \tag{S31}$$

$$q(\boldsymbol{\theta}^* \mid \boldsymbol{\theta}_k) = N(\boldsymbol{\theta}_k, diag(w_i^2)) \tag{S32}$$

   This distribution is centered around the current state, with covariance matrix $diag(w_i^2)$, where w_i is the proposal width and is a pre-selected constant for each parameter i.

3. Evaluate the likelihood of the candidate and current states by solving $\boldsymbol{g}(\boldsymbol{\theta})$, which as mentioned in the main text is the solution to the system of non-linear equations of the model and returns J_M and y_{BDO}. For binary feeds, this can be arranged into a quadratic form (equations 16-19 of the main text) and after converting to the appropriate units yields the predicted values of $\boldsymbol{Y}$ (J_{LMH} and $w_{perm,BDO}$).

4. Calculate the Hasting's ratio for the candidate state, $\alpha(\boldsymbol{\theta}^*, \boldsymbol{\theta}_k)$.

$$\alpha(\boldsymbol{\theta}^*, \boldsymbol{\theta}_k) = \frac{p(\boldsymbol{\theta}^* \mid \boldsymbol{Y}) q(\boldsymbol{\theta}^* \mid \boldsymbol{\theta}_k)}{p(\boldsymbol{\theta}_k \mid \boldsymbol{Y}) q(\boldsymbol{\theta}_k \mid \boldsymbol{\theta}^*)} = \frac{p(\boldsymbol{Y} \mid \boldsymbol{\theta}^*) p(\boldsymbol{\theta}^*) q(\boldsymbol{\theta}^* \mid \boldsymbol{\theta}_k)}{p(\boldsymbol{Y} \mid \boldsymbol{\theta}_k) p(\boldsymbol{\theta}_k) q(\boldsymbol{\theta}_k \mid \boldsymbol{\theta}^*)} \tag{S33}$$

   Assuming an uninformative prior, $p(\boldsymbol{\theta}) \propto 1$, and given that the proposal distribution is normal and thus symmetric, $q(\boldsymbol{\theta}^* \mid \boldsymbol{\theta}_k) = q(\boldsymbol{\theta}_k \mid \boldsymbol{\theta}^*)$, α reduces to $\frac{p(\boldsymbol{Y} \mid \boldsymbol{\theta}^*)}{p(\boldsymbol{Y} \mid \boldsymbol{\theta}_k)}$. The Metropolis probability acceptance, $A(\boldsymbol{\theta}^*, \boldsymbol{\theta}_k)$, is then determined by,

$$A(\boldsymbol{\theta}^*, \boldsymbol{\theta}_k) = min\left[1, \frac{p(\boldsymbol{Y} \mid \boldsymbol{\theta}^*)}{p(\boldsymbol{Y} \mid \boldsymbol{\theta}_k)}\right] \tag{S34}$$

   where $p(\boldsymbol{Y} \mid \boldsymbol{\theta}^*)$ can be readily computed from equations (S26) and (S27).

5. Draw a random sample from a uniform distribution, $\beta \sim U(0,1)$.

6. Select the new current state, $\boldsymbol{\theta}_{k+1}$, based on the following criteria

$$\boldsymbol{\theta}_{k+1} = \begin{cases} \boldsymbol{\theta}^*, & \beta \le A \\ \boldsymbol{\theta}_k, & \beta > A \end{cases} \tag{S35}$$

This means that the candidate state is rejected if it fails to improve the likelihood with probability A and $\boldsymbol{\theta}_k$ is retained. Otherwise $\boldsymbol{\theta}^*$ is accepted and becomes the new state.

7. Return to step 2 and repeat throughout n iterations.

This basic outline of MH provides a simple yet robust method for parameter estimation. It can be computationally expensive if the predictor $\boldsymbol{g}(\boldsymbol{\theta})$ is complex and more advanced variations of the algorithm would be needed to help reduce the cost. In the present study, $\boldsymbol{g}(\boldsymbol{\theta})$ is inexpensive to evaluate, making this version of the MH algorithm well-suited for sampling $p(\boldsymbol{\theta} \mid \boldsymbol{Y})$. Algorithm S1 summarizes the implementation of the MH algorithm.

Algorithm S1 Bayesian Inference via Metropolis-Hastings MCMC

Input: Initial state θ_0, proposal widths w_i, number of iterations n
Output: Markov chain $\{\theta_0, \theta_1, \ldots, \theta_n\}$
Set initial state θ_0 ▷ Refer to Step 1
for $k = 0$ to $n-1$ **do**
  Propose a new candidate state $\theta^* \sim q(\theta^* \mid \theta_k)$ ▷ Refer to Step 2
  Calculate y_{BDO} and J_M ▷ Refer to Step 3
  Compute the Hastings ratio $\alpha(\theta^*, \theta_k)$ ▷ Refer to Step 4
  Draw $\beta \sim U(0,1)$
  if $\beta \le \alpha(\theta^*, \theta_k)$ **then**
    Accept the candidate: $\theta_{k+1} \leftarrow \theta^*$
  else
    Reject the candidate: $\theta_{k+1} \leftarrow \theta_k$
  end if
end for
Return: $\{\theta_0, \theta_1, \ldots, \theta_n\}$

S3.3 Effect of Prior Distribution

The effect of the prior on the posterior was also evaluated. In step 4 of the algorithm, the prior was assumed to be uninformative, $p(\boldsymbol{\theta}) \propto 1$. However, this doesn't need to be the case. **Figure** S5 shows the effect of the prior on the posterior density. For the uniform prior we used the upper ($\boldsymbol{ub}$) and lower ($\boldsymbol{lb}$) bounds of the marginal 95% confidence interval shown in Table 3 of the main text, $p(\boldsymbol{\theta}) \sim Unif(\boldsymbol{lb}, \boldsymbol{ub})$. For Gaussian 1, we assumed a normal distribution, $p(\boldsymbol{\theta}) \sim N(\boldsymbol{\theta}_{\mathrm{GLSR}}, \boldsymbol{\Sigma}_{\mathrm{GLSR}})$, with mean equal to

the deterministic estimate via GLSR ($\boldsymbol{\theta}_{\mathrm{GLSR}}$) in **Table 3** of the main text and covariance ($\boldsymbol{\Sigma}_{\mathrm{GLSR}}$) from the frequentist estimate shown in. For Gaussian 2, we assumed a normal distribution centered at the initial guess of the GLSR method, $\boldsymbol{\theta}_0$. As an initial guess for GLSR, we used simplifying estimates based on preliminary experiments, heuristics or values reported in literature. **Table S9** shows this initial guess. For $Q_{0,water}$ we used the value of permeance calculated from the control experiment (100% water feed at 353 K) shown in Table, while $Q_{0,BDO}$ was assumed to be lower but in the same order of magnitude. This is consistent with previous studies analyzing pervaporation of organic/water feeds at similar concentrations. The initial guesses of $E_{a,water}$ and $A_{conc,BDO}$ were based on the slopes of the linearized plots (Figure 4 of the main text). For the covariance of this distribution, we assumed a diagonal matrix with variance entries equal to 0.30 of the mean value ($\boldsymbol{\Sigma} = \mathrm{diag}(0.30 \times \boldsymbol{\theta}_0)$). This gives a prior distribution given by $p(\boldsymbol{\theta}) \sim N\big(\boldsymbol{\theta}_0, \mathrm{diag}(0.30 \times \boldsymbol{\theta}_0)\big)$.

As shown, for the uninformative, uniform and Gaussian 1 prior, the results is almost identical, denoting that for well-informed priors. For Gaussian 1, the confidence region is slightly narrower, showing the prior can have an important effect. For Gaussian 2, however, the estimate is significantly different. This is a poorly informed and concentrated prior that is not flexible to new information. Thus, the small and uncertain dataset of this work is not enough evidence to overturn this prior.

S3.4 Bounds on Variables and Multi-start Solver for the GLSR Method

Bounds to solve the regression problem through the GLSR method were also selected through heuristics and literature values. **Table S9** shows the upper and lower bounds for the mode parameters. For the pre-exponential permeance terms (both water and BDO), upper and lower bounds were set one order of magnitude above and two orders of magnitude below the initial guess, respectively. This is a broad enough search space for the solver to explore and allow identification of these two parameters. It also encompasses the values reported for the pre-exponential permeance term for organic/water mixtures. For $E_{a,water}$, initial a negative value was estimated, however we allowed for a wide range (±100 kJ/mol) to also include positive activation energy values (which are more common). Finally, for $A_{conc,BDO}$,

which is empirical, bounds were selected arbitrarily but remain consistent with the range of values reported in previous studies.

IPOPT is an interior-point solver and does not guarantee a global solution. This might lead to local minima, so we used a Pyomo contribution package called "Multistart Solver" which addresses this challenge[13]. This package solves the minimization problem at multiple starting points using a random restarting strategy and selects the best solution. We didn't observe any difference in the solution for as many as 50 different starting points

S3.5 Frequentist Uncertainty Quantification

As mentioned in the main text, we also estimated the confidence intervals for the generalized least square regression (GLSR) method, which corresponds to a frequentist approach for uncertainty quantification. The following expression from Bates and Watts was used to estimate the marginal confidence region of $\theta_{GLSR,i}$, where $\boldsymbol{\theta}_{GLSR}$ is the parameter vector estimated from the GLSR method and $\theta_{GLSR,i}$ is the ith element of this vector:

$$\theta_{GLSR,i} \pm t_{N-P,\frac{\alpha}{2}} \cdot s \cdot \sqrt{2\{\boldsymbol{H}^{-1}\}_{ii}} \quad \text{(S36)}$$

Here, $t_{N-P,\frac{\alpha}{2}}$ is the critical value from the Student's t-distribution with N – P degrees of freedom at a significance level of $\frac{\alpha}{2}$. N is the number of observations and P is the number of parameters. Since there are 7 experiments and 2 measurements responses per experiment, N = 14. There are 4 parameters that are estimated so P = 4. To obtain a 95% confidence interval, we set $\alpha = 0.05$. This gives a t-distribution critical value of $t_{10,0.025} = 2.2281$. Furthermore, s is a scale factor given by:

$$s = \left(\frac{J(\boldsymbol{\theta}_{\text{GLSR}})}{N-P}\right)^{0.5} \quad \text{(S37)}$$

Where $J(\boldsymbol{\theta}_{\text{GLSR}})$ is the loss function of the GLRS, $J(\boldsymbol{\theta})$, evaluated at $\boldsymbol{\theta}_{GLSR}$. As mentioned in the main text, $g(\boldsymbol{\theta})$ is the model prediction and $\boldsymbol{Y}$ is a vector containing the experimental measurements (flux, LMH, and BDO permeate composition).

$$J(\boldsymbol{\theta}) = (g(\boldsymbol{\theta}) - \boldsymbol{Y})^T \boldsymbol{\Sigma}^{-1} (g(\boldsymbol{\theta}) - \boldsymbol{Y}) \quad \text{(S38)}$$

$\mathbf{\Sigma}$ is the measurement error covariance matrix, which as mentioned was obtained from replicate experimental samples of flux and BDO permeate composition. We assumed $\mathbf{\Sigma}$ is a diagonal matrix and standard deviations of $\sigma_{LMH} = 0.01\ L\ m^{-2}\ h^{-1}$ and $\sigma_{perm,BDO} = 0.0075$, this gives:

$$\mathbf{\Sigma} = \begin{bmatrix} 1x10^{-4} & 0 \\ 0 & 5.625x10^{-5} \end{bmatrix} \tag{S39}$$

This results in $s = 2.798$. Lastly, H is the Hessian of $J(\boldsymbol{\theta})$ evaluated at $\boldsymbol{\theta}_{GLSR}$. Given that $J(\boldsymbol{\theta})$ is an explicit expression, we analytically computed $\boldsymbol{H}$ in Equation (S40) using the symbolic differentiation module from pyomo (pyomo.core.expr.calculus.derivatives.differentiate). As mentioned in the main text, the measurement responses can be obtained from the quadratic formula, and any other symbolic differentiation tool could have also been used in this problem. The computed $\boldsymbol{H}$ is shown in Equation (S41), and the corresponding units of each entry are shown in Equation (S42). $\{\boldsymbol{H}^{-1}\}_{ii}$ is the ith element in the diagonal of the inverse of the matrix.

$$\boldsymbol{H} = \begin{bmatrix} \frac{\partial^2 J}{\partial^2 Q_{0,water}} & \frac{\partial J}{\partial Q_{0,water}\partial Q_{0,BDO}} & \frac{\partial J}{\partial Q_{0,water}\partial E_{a,water}} & \frac{\partial J}{\partial Q_{0,water}\partial A_{conc,BDO}} \\ \frac{\partial J}{\partial Q_{0,BDO}\partial Q_{0,water}} & \frac{\partial^2 J}{\partial^2 Q_{0,BDO}} & \frac{\partial J}{\partial Q_{0,BDO}\partial E_{a,water}} & \frac{\partial J}{\partial Q_{0,BDO}\partial A_{conc,BDO}} \\ \frac{\partial J}{\partial E_{a,water}\partial Q_{0,water}} & \frac{\partial J}{\partial E_{a,water}\partial Q_{0,BDO}} & \frac{\partial^2 J}{\partial^2 E_{a,water}} & \frac{\partial J}{\partial E_{a,water}\partial A_{conc,BDO}} \\ \frac{\partial J}{\partial A_{conc,BDO}\partial Q_{0,water}} & \frac{\partial J}{\partial A_{conc,BDO}\partial Q_{0,BDO}} & \frac{\partial J}{\partial A_{conc,BDO}\partial E_{a,water}} & \frac{\partial^2 J}{\partial^2 A_{conc,BDO}} \end{bmatrix} \tag{S40}$$

$$\boldsymbol{H} = \begin{bmatrix} 7.50\ x\ 10^{17} & 4.33\ x\ 10^{-18} & 3.46\ x\ 10^{5} & -1.95\ x\ 10^{9} \\ 4.33\ x\ 10^{18} & 1.65\ x\ 10^{18} & -2.39\ x\ 10^{5} & 7.57\ x\ 10^{9} \\ 3.46\ x\ 10^{5} & -2.39\ x\ 10^{5} & 3.63\ x\ 10^{7} & -1.01\ x\ 10^{-3} \\ -1.95\ x\ 10^{9} & 7.57\ x\ 10^{9} & -1.01\ x\ 10^{-3} & 4.03\ 10^{1} \end{bmatrix} \tag{S41}$$

$$\textit{units of H} = \begin{bmatrix} \text{mol}^2\ \text{m}^{-4}\ \text{s}^{-2}\ \text{Pa}^{-2} & \text{mol}^2\ \text{m}^{-4}\ \text{s}^{-2}\ \text{Pa}^{-2} & \text{kJ}\ \text{m}^{-2}\ \text{s}^{-1}\ \text{Pa}^{-1} & \text{mol}\ \text{m}^{-2}\ \text{s}^{-1}\ \text{Pa}^{-1} \\ \text{mol}^2\ \text{m}^{-4}\ \text{s}^{-2}\ \text{Pa}^{-2} & \text{mol}^2\ \text{m}^{-4}\ \text{s}^{-2}\ \text{Pa}^{-2} & \text{kJ}\ \text{m}^{-2}\ \text{s}^{-1}\ \text{Pa}^{-1} & \text{mol}\ \text{m}^{-2}\ \text{s}^{-1}\ \text{Pa}^{-1} \\ \text{kJ}\ \text{m}^{-2}\ \text{s}^{-1}\ \text{Pa}^{-1} & \text{kJ}\ \text{m}^{-2}\ \text{s}^{-1}\ \text{Pa}^{-1} & \text{kJ}\ \text{mol}^{-2} & \text{kJ}\ \text{mol}^{-1} \\ \text{mol}\ \text{m}^{-2}\ \text{s}^{-1}\ \text{Pa}^{-1} & \text{mol}\ \text{m}^{-2}\ \text{s}^{-1}\ \text{Pa}^{-1} & \text{kJ}\ \text{mol}^{-1} & - \end{bmatrix} \tag{S42}$$

The parameter covariance matrix, $\mathbf{\Omega}$, can be estimated as follows:

$$\mathbf{\Omega} = 2s^2\boldsymbol{H}^{-1} \tag{S43}$$

S4. Mathematical Models

S4.1 Derivation of mass and energy balances

in the main text shows the representation of a membrane module. A mass balance around the differential area element of size ΔA gives the following relation, where $\dot{n}$ is the molar flow rate and J_M is the total molar flux.

$$\dot{n}|_A = \dot{n}|_{A+\Delta A} + J_M \Delta A \quad \text{(S44)}$$

$$\frac{\dot{n}|_A - \dot{n}|_{A+\Delta A}}{\Delta A} = J_M \quad \text{(S45)}$$

$$\frac{d\dot{n}}{dA} = -J_M \quad \text{(S46)}$$

Similarly, we can carry the component mass balance, where x_i and y_i are the molar compositions of component i in the liquid and vapor phases, respectively.

$$\frac{(\dot{n}x_i)|_A - (\dot{n}x_i)|_{A+\Delta A}}{\Delta A} = J_M y_i \quad \text{(S47)}$$

$$\frac{d(\dot{n}x_i)}{dA} = -J_M y_i \quad \text{(S48)}$$

$$\frac{d\dot{n}}{dA}x_i + \dot{n}\frac{dx_i}{dA} = -J_M y_i \quad \text{(S49)}$$

Replacing $\frac{d\dot{n}}{dA}$ for the expression in equation (S46) gives

$$-J_M x_i + \dot{n}\frac{dx_i}{dA} = -J_M y_i \quad \text{(S50)}$$

$$\frac{dx_i}{dA} = -J_M \frac{y_i - x_i}{\dot{n}} \quad \text{(S51)}$$

We can then conduct the energy balance.

$$(\dot{n}H)|_A = (\dot{n}H)|_{A+\Delta A} + (J_M \Delta A)H^{perm} \quad \text{(S52)}$$

Here, H is the specific enthalpy of the mixture, which can be obtained from specific enthalpy of the pure components, H_i, and assuming ideal mixing. H^{perm} is the specific enthalpy of the permeate.

$$H = \sum_{k=1}^{N} x_k H_k \tag{S53}$$

$$\frac{\left(\sum \dot{n} x_k H_k\right)|_A - \left(\sum \dot{n} x_k H_k\right)|_{A+\Delta A}}{\Delta A} = J_M H^{perm} \tag{S54}$$

$$\frac{d\left(\sum \dot{n} x_k H_k\right)}{dA} = \sum \frac{d(\dot{n} x_k H_k)}{dA} = -J_M H^{perm} \tag{S55}$$

$$\sum \left[\frac{d(\dot{n} x_k)}{dA} H_k + (\dot{n} x_k) \frac{dH_k}{dA}\right] = -J_M H^{perm} \tag{S56}$$

Replacing $\frac{d(\dot{n}x_k)}{dA}$ with the term in equation (S48) gives

$$\sum \left(-J_M y_k H_k + \dot{n} x_k \frac{dH_k}{dA}\right) = -J_M H^{perm} \tag{S57}$$

$$\sum \dot{n} x_k \frac{dH_k}{dA} = -J_M \left(H^{perm} - \sum y_k H_k\right) \tag{S58}$$

The pure component specific enthalpy as a function of temperature, $H_i(T)$, can be obtained using the following relation. T_0 is a reference or datum temperature, which makes $H_i(T_0)$ a constant.

$$H_i(T) = H_i(T_0) + \int_{T_0}^{T} \hat{C}_{p,i} dT \tag{S59}$$

The constant-pressure heat capacity, $\hat{C}_{p,i}$, is assumed to be invariant with temperature, which is a common assumption for liquids. This leads to the following expressions and simplification for $\frac{dH_i}{dA}$.

$$\int_{T_0}^{T} \hat{C}_{p,i} dT = \hat{C}_{p,i}(T - T_0) \tag{S60}$$

$$\frac{dH_i}{dA} = \hat{C}_{p,i} \frac{dT}{dA} \tag{S61}$$

The above expressions assume no phase change. However, the permeate is obtained from vaporization of the mixture in the feed/retentate side. We include the enthalpy of vaporization at the temperature in the permeate side, $\Delta H_i^{vap}\left(T_{perm}\right)$, where T_{perm} is the dew point temperature of the permeate at the vacuum pressure P_p. We still assume ideal mixing for the permeate mixture.

$$H_i^{perm} = H_i(T_0) + \hat{C}_{p,i}(T_{perm} - T_0) + \Delta H_i^{vap}(T_{perm}) \tag{S62}$$

$$H^{perm} = \sum y_k H_k^{perm} \tag{S63}$$

Collecting terms leads to the following expression, where $H^{perm}(T_{perm})$ is obtained from equations (S62) and (S63), and $H_k(T)$ from equations (S59) and (S60).

$$H^{perm}(T_{perm}) - \sum y_k H_k(T) = \sum y_k[\hat{C}_{p,k}(T_{perm} - T) + \Delta H_k^{vap}] \tag{S64}$$

This can be plugged back into equation (S58) to obtain the final energy balance.

$$\dot{n}\left(\sum x_k \hat{C}_{p,k}\right)\frac{dT}{dA} = -J_M \sum y_k[\hat{C}_{p,k}(T_{perm} - T) + \Delta H_k^{vap}] \tag{S65}$$

S4.2 Solution to the system of differential algebraic equations

As explained in the main text, the mass and energy balances along with the boundary conditions yield a system of differential algebraic equations (DAEs). As a reminder, in this system we can distinguish between differential, $\boldsymbol{z}$, and algebraic, $\boldsymbol{v}$, variables.

$$\dot{n}^j, x_i^j, T^j = \boldsymbol{z}(A; \boldsymbol{\theta}) \tag{S66}$$

$$J_M^j, y_i^j, T_{perm}^j = \boldsymbol{v}(A; \boldsymbol{\theta}) \tag{S67}$$

The system of DAEs can now be solved. As shown in Algorithm S2 we employed an explicit method (Euler's method). The only parameter that needs to be defined is $NFET$, the number of finite elements. We fine-tuned this value and found that $NFET = 10{,}000$ offers a good trade-off between accuracy and computational time.

Algorithm S2 Membrane Module Simulation Algorithm

Input: $NFET$, $A^j_{m,\max}$, $x_{\text{final,BDO}}$, z^0, $T^j_{\text{feed}}\ \forall j$

Output: Numerical solution of z and v for all module iterations

$dA \leftarrow \frac{A^j_{m,\max}}{NFET}$

$z \leftarrow z^0$ ▷ Set feed conditions for the first module

$j \leftarrow 1$ ▷ Initialize module counter

while $x_{\text{BDO}} > x_{\text{final,BDO}}$ **do**

  $\dot{n}^j_{\text{permeate}} \leftarrow 0$ ▷ Initialize permeate flux for module j

  for $i = 1$ **to** $NFET$ **do**

    if $x_{\text{BDO}} \leq x_{\text{final,BDO}}$ **then**

      break ▷ Exit if target concentration is reached

    end if

    Solve for v ▷ Equations 16-19 main text

    if $J_M < 0$ **then**

      break ▷ Exit if flux becomes negative

    end if

    $\dot{n}^j_{\text{permeate}} \leftarrow \dot{n}^j_{\text{permeate}} + \sum(J_i \times dA)$ ▷ Update permeate flow

    Solve for dz using Eqs. 20–22

    $z \leftarrow z + dz$ ▷ Update state variables

  end for

  $j \leftarrow j+1$ ▷ Advance to the next module

end while

return z, v

S4.3 Derivation of the quadratic equation to solve for y_{BDO}, J_{water} and J_M

First, we start with the general underlying permeance expressions for each component as described in the main text. We also need the component balance equation.

$$y_i J_M = Q_i \left(\hat{f}^L_{feed,i} - y_i P_p \right) \tag{S68}$$

$$\sum y_i = 1 \tag{S69}$$

Next, we specify the expressions for the components in our process, BDO and water

$$y_{BDO} J_M = Q_{BDO} \left(\hat{f}^L_{feed,BDO} - y_{BDO} P_p \right) \tag{S70}$$

$$y_{water} J_M = Q_{water} \left(\hat{f}^L_{feed,water} - y_{water} P_p \right) \tag{S71}$$

$$y_{BDO} + y_{water} = 1 \tag{S72}$$

This system of three equations can be reduced to two by using the component balance

$$y_{BDO} J_M = Q_{BDO} \left(\hat{f}^L_{feed,BDO} - y_{BDO} P_p \right) \tag{S73}$$

$$(1 - y_{BDO}) J_M = Q_{water} \left[\hat{f}^L_{feed,water} - (1 - y_{BDO}) P_p \right] \tag{S74}$$

Solve for J_M

$$J_M = \frac{Q_i \left[\hat{f}^L_{feed,water} - (1 - y_{BDO}) P_p \right]}{(1 - y_{BDO})} \tag{S75}$$

Replace J_M to further reduce the system from two equations to a single equation

$$(y_{BDO}) \frac{Q_{water} \left[\hat{f}^L_{feed,water} - (1 - y_{BDO}) P_p \right]}{(1 - y_{BDO})} = Q_{BDO} \left(\hat{f}^L_{feed,BDO} - y_{BDO} P_p \right) \tag{S76}$$

Reorder the variables

$$(y_{BDO}) Q_{water} \left[\hat{f}^L_{feed,water} - (1 - y_{BDO}) P_p \right] = (1 - y_{BDO})\, Q_{BDO} \left(\hat{f}^L_{feed,BDO} - y_{BDO} P_p \right) \tag{S77}$$

Expand

$$Q_{water} \left(y_{BDO} \hat{f}^L_{feed,water} - y_{BDO} P_p + y^2_{BDO} P_p \right) = Q_{BDO} \left(\hat{f}^L_{feed,BDO} - y_{BDO} \hat{f}^L_{feed,BDO} - y_{BDO} P_p + y^2_{BDO} P_p \right) \tag{S78}$$

Group variables to obtain the final quadratic expression shown in the main text

$$y_{BDO}^2\left[P_p(Q_{water} - Q_{BDO})\right] + y_{BDO}\left[Q_{BDO}\hat{f}_{feed,BDO}^{L} + Q_{water}\hat{f}_{feed,water}^{L} + P_p(Q_{BDO} - Q_{water})\right] - Q_{BDO} \tag{S79}$$

S4.4 Economic model – equipment sizing

S4.4.1 Membrane Modules

To estimate the dimensions of each module we first need to make assumptions regarding the individual membrane fibers. A common approach is to assume a fixed value for diameter, d_{fiber}, and length, L_{memb}, of the fibers. For d_{fiber} we assumed a value of 0.85 mm, which is approximately the same as the diameter of the fibers in the experimental set up. For L_{memb} we used a value of 2.5 m, which is in the upper end of the range of typical fiber length values of industrial membranes. This was also assumed to be the length of the membrane module. With this information we estimated the diameter of the module based on available correlations.

In practice, each stage is composed of parallel modules. In this setup the flow rate of the stream entering a stage is split and sent to each module. This helps reduce the size of each individual module and achieve practical diameters. Large module diameters can be challenging to manufacture and install, and by splitting the total membrane area of the stage into submodules, we can maintain manageable diameter values. A_{stage} is the total membrane area of a stage in the cascade, which is the summation of each individual module, A_{module}. For hollow fiber membranes, 10,000 – 1,000,000 fibers can be packed in each module. We assumed the upper limit of A_{module} is 1,000 m^2. As mentioned in the main text, the area of each stage in the proposed cascade, A_{stage}, was around 3,200 m^2, so we assumed $A_{module} = 800\ m^2$. This gives four parallel modules per stage, $N_{modules} = 4$.

$$A_{stage} = total\ membrane\ area\ of\ a\ stage\ (m^2)$$

$$A_{module} = area\ of\ a\ membrane\ module\ (m^2)$$

$$N_{modules} = number\ of\ modules\ per\ stage$$

$$A_{module} = \frac{A_{module}}{N_{modules}} \tag{S80}$$

A_{module} is obtained for each module by solving the governing equations. Thus, N_{fibers} is the only variables that still needs to be estimated.

$$N_{membs} = number\ of\ membrane\ fibers\ packed\ in\ a\ given\ module$$

$$d_{fiber} = outer\ diameter\ of\ a\ membrane\ fiber\ (m)$$

$$L_{memb} = length\ of\ a\ membrane\ fiber\ (m)\ (same\ as\ length\ of\ the\ module)$$

$$A_{module} = N_{membs} \pi d_{fiber} L_{memb} \tag{S81}$$

$$N_{fibers} = \frac{\pi d_{fiber} L_{memb}}{A_{module}} \tag{S82}$$

This is all the information needed to estimate the diameter of the module. As proposed by Constantino et al.[14], the module containing the membrane fibers can be sized using the following expression. Similar to the design from Constantino et al. we assume $CL = 0.87$ (equilateral triangular pitch), $CTP = 0.93$ (one tube pass) and $PR = 1.25$ (pitch ratio assuming a packing efficiency of 58%).

$$d_{module} = module\ outer\ diameter\ (m)$$

$$CL = tube\ layout\ constant\ (-)$$

$$CTP = tube\ count\ constant$$

$$PR = pitch\ ratio$$

$$d_{module} = 0.637\left[\left(\frac{CL}{CTP}\right)\frac{A_{module}(PR)^2 d_{fiber}}{L_{memb}}\right]^{\frac{1}{2}} \tag{S83}$$

S4.4.2 Pre-heaters

As mentioned, prior to each module there is a heat exchanger that heats the feed to the inlet temperature T_{feed}. The area of these exchangers is estimated as follows

$$\dot{Q} = absolute\ value\ of\ heat\ duty\ (W)$$

$$U = overall\ heat\ transfer\ coefficient\ (W\ m^{-2}\ K^{-1})$$

$$\Delta T_{log} = log - mean\ temperature\ difference\ (K)$$

$$T_{\frac{hot}{cold},\frac{out}{in}} = inlet\ or\ outler\ temperature\ of\ the\ hot\ or\ cold\ streams\ (K)$$

$$A_{HX} = heat - tranfer\ area\ (ft^2)$$

$$A_{HX} = \frac{\dot{Q}}{\mathrm{U}\Delta T_{log}} \times \frac{(3.21\ ft)^2}{m^2} \tag{S84}$$

$$\Delta T_{log} = \frac{\Delta T_2 - \Delta T_1}{\ln\left(\frac{\Delta T_2}{\Delta T_1}\right)} \tag{S85}$$

$$\Delta T_1 = T_{hot,out} - T_{cold,in} \tag{S86}$$

$$\Delta T_2 = T_{hot,in} - T_{cold,out} \tag{S87}$$

For the pre-heater in stage j of the cascade, we assume the feed stream, $\dot{n}^j_{feed}$, needs to be raised from the outlet temperature of the previous stage $T^{j-1}|_{A=A^{j-1}_{module}}$ to the inlet temperature T^j_{feed}.

$$\dot{Q}^j = \dot{Q}^j_{heat} = \dot{n}^j_{feed}\left(\sum_k x^j_{k,feed}\hat{C}_{p,k}\right)\left(T^j_{feed} - T^{j-1}|_{A=A^{j-1}_{module}}\right) \tag{S88}$$

$$T^j_{cold,in} = T^{j-1}|_{A=A^{j-1}_{module}} \tag{S89}$$

$$T^j_{cold,out} = T^j_{feed} \tag{S90}$$

The only exception is the first stage where we the stream entering the pre-heater comes from the SMB+distillation pre-concentration step[15].

$$T^1_{cold,in} = T_{SMB+dist} \tag{S91}$$

For the hot stream, we assume low pressure steam at 506 K and 10 bar.

$$T_{hot,out} = T_{hot,in} = T_{LPS} = 506\ K \tag{S92}$$

$$U_{heating} = 1900\ W\ m^{-2}\ K^{-1} \tag{S93}$$

S4.4.3 Centrifugal pumps

We chose centrifugal pumps to increase the pressure of the retentate leaving a membrane module before it enters a new stage. The sizing factor, $\boldsymbol{S}$, is given by the pressure drop and the pump head

(Seider et al. Eq 22.13). For these hollow fiber membranes, we propose that the liquid feed flows through the tube side of the submodule, while the vapor permeate flows through the shell side. For the liquid, the pressure drop is estimated from Hagen-Poiseuille's law (this expression can be applied since flow remains laminar Re<100). As mentioned, the fiber and module dimensions (diameter and length) are pre-specified.

$$\Delta P = pressure\ drop\ in\ a\ module\ (Pa)$$

$$\mu = \ mixture\ viscosity\ \left(\frac{\frac{kg}{m}}{s}\right)$$

$$L_{mod} = module\ length\ (m)$$

$$F = volumetric\ flow\ rate\ through\ a\ module\ \left(\frac{gal}{min}\right)$$

$$= volumetric\ flow\ rate\ through\ a\ submodule\ (m^3/s)$$

$$f = \frac{F}{N_{modules}} \times \frac{1\frac{m^3}{s}}{15850\ gal/min} \quad \text{(S94)}$$

$$\Delta P = \frac{128\mu L_{mod}\left(\frac{f}{N_{fibers}}\right)}{\pi d_{fiber}^4} \quad \text{(S95)}$$

Once the hydraulic diameter and the pressure drop is obtained the pump can be sized. The following expressions were used:

$$H = pump\ head\ (ft)$$

$$\rho = mixture\ density\ \left(\frac{kg}{m^3}\right)$$

$$g = gravity\ \left(9.81\frac{m}{s^2}\right)$$

$$g = gravity\ \left(9.81\frac{m}{s^2}\right)$$

$$S = pump\ size\ factors\ \left(\frac{gal}{\min\ ft^{0.5}}\right)$$

$$H = \frac{\Delta P}{\rho g} \times \frac{3.21\,ft}{m} \tag{S96}$$

$$S = FH^{0.5} \tag{S97}$$

In addition, the motors of these pumps also need to be sized. The sizing factor is the motor power consumption, which can be estimated as follow (Seider et al. Eqs 22.16 – 22.19, some multiplying factors were modified to account for unit conversion).

$$\dot{W} = required\ pump\ work\ (Watt)$$

$$\eta_p = Brake\ horsepower\ efficiency$$

$$\eta_m = Electric\ motor\ efficiencyg = gravity\ \left(9.81\frac{m}{s^2}\right)$$

$$P_b = pump\ brake\ horsepower\ (Hp)$$

$$P_c = motor\ power\ consumption\ (Hp)$$

$$\eta_p = 0.316 + 0.24015 \times \ln(F) - 0.01199 \times \ln(F)^2 \tag{S98}$$

$$W_{pump} = \Delta P \left(F \times \frac{1\frac{m^3}{s}}{15850\ gal/min} \right) \tag{S99}$$

$$P_b = \frac{\dot{W}}{\eta_p} \times \frac{745.7\ Hp}{Watt} \tag{S100}$$

$$\eta_m = 0.8 + 0.0319 \times \ln(P_b) - 0.000182 \times \ln(P_b)^2 \tag{S101}$$

$$P_c = \frac{P_b}{\eta_m} \tag{S102}$$

S4.4.4 Condenser

We assume the vapor permeate from each stage, $\dot{n}^j_{perm}$, is collected into a single stream, requiring only one condenser. Equations (S84) – (S87) are still applicable to estimate the area of this condenser. Since the permeate composition is nearly uniform (>99wt.% water) across all stages, we assume the temperature of the single permeate stream, T_{perm}, is the dew point temperature of pure water at the fixed vacuum pressure. Furthermore, we assume no temperature change occurs during condensation

(only phase change), and the absolute value of cooling duty is equal to the enthalpy of vaporization at the permeate temperature, $\Delta H_i^{vap}(T_{perm})$.

$$\dot{Q} = \dot{Q}_{cool} = \left(\sum \dot{n}_{perm}^{k}\right) \Delta H_i^{vap}(T_{perm}) \tag{S103}$$

$$T_{hot,in} = T_{hot,\,out} = T_{perm} \tag{S104}$$

We assumed chilled water from the biorefinery is the cold stream that can provide the cooling duty. The inlet and outlet temperature values were obtained from the original biorefinery design. The overall heat transfer coefficient, $U_{cooling}$, was obtained from Seider et al, Heuristic 56.

$$U_{cooling} = 567\ W\ m^{-2}\ K^{-1} \tag{S105}$$

$$T_{cold,in} = 277\ K \tag{S106}$$

$$T_{cold,out} = 288\ K \tag{S107}$$

S4.4.5 Vacuum pump

Lastly, based on the cascade design, only one vacuum pump is required. We assumed a single-stage liquid ring pump is capable of providing vacuum pressures as low as 7 kPa. Using the correlations from Seider et al.[16], the sizing variable is the suction rate, S_{vac}. This includes ambient air leakage leaving the equipment and the flow rate of components in the composite permeate that are in equilibrium with this air stream. The air leakage rate can be estimated as follows from Seider et al. Eq 22.73[16]. The evacuation volume includes only the pieces of equipment operating under vacuum. In this case the permeate stream flows in the tube side of the modules, so the evacuation volume corresponds to the total inner volume of the membrane fibers. A multiplying factor of 1.15 was applied to V_{vac} to account for valves, joints, gaskets and other components prone to air leakage. The empirical correlation of S_{vac} as a function of W_{air} was developed based on data obtained from repeated flash tank simulations performed in Aspen Plus at $P_p = 7\ kPa$. The range of W_{air} was between 7-20 lb/hr, more details on how we developed the correlation for S_{vac} can be found in Section S5.1.

$$V_{module}^{j} = Inner\ fiber\ tube\ volume\ of\ the\ j^{th}\ stage\ module\ (m^3)$$

$$W_{air} = air\ leakage\ rate\ \left(\frac{lb}{hr}\right)$$

$$P_{vac} = aboslute\ operating\ pressure\ of\ the\ vacuum\ system\ (torr)$$

$$S_{vac} = suction\ rate\ of\ air\ and\ other\ components\ \ (ft^3/min)$$

$$V_{vac} = evacuation\ volume\ \left(ft^3\right)$$

$$V_{module}^{j} = \frac{N_{fiber}\left(\pi d_{fiber}^{2} L_{memb}\right)}{4} \quad \text{(S108)}$$

$$V_{vac} = \ 1.15 \sum V_{module}^{k} \times \frac{(3.21\ ft)^3}{m^3} \quad \text{(S109)}$$

$$W_{air} = 5 + \left(0.0298 + 0.03088 \times \ln(P_{vac}) - 0.00057333 \times \ln{(P_{vac})^2}\right) V_{vac}^{0.67} \quad \text{(S110)}$$

$$S_{vac} = -7.70x10^{-3} \times W_{air}^{2} + 9.28 \times W_{air} + 47.6 + 73.0 \times \ln(W_{air}) \quad \text{(S111)}$$

S4.5 Economic model – equipment costing, purchase equipment cost ($)

Based on the sizing factor from the previous section we obtain the total equipment purchase cost, C_p ($), using the following correlations.

S4.5.1 Membrane module cost

We assume the unit area cost, $C_{memb}\left(\frac{\$}{m^2}\right)$, includes the cost of the dense PVDF membrane fiber material as well as the module containing the fibers.

$$C_{p,\,modules} = C_{p,memb} \sum A_{memb}^{k} \quad \text{(S112)}$$

S4.5.2 Pre-heaters

Depending on the size of the exchanger (measured by its heat-transfer area, A_{HX}, ft^2) it could either be a spiral tube (small unit) or spiral plate (larger unit). Correlations were obtained from Seider et al.[16] Table 22.32.

$$C_{p,pre-heater}^{j} = \begin{cases} \exp\left\{8.0757 + 0.4343[\ln(A_{HX})] + 0.03812[\ln(A_{HX})]^2\right\}, & A_{HX} \leq 20ft^2 \\ 6200 \times A^{0.42}, & A_{HX} > 20ft^2 \end{cases} \quad \text{(S113)}$$

S4.5.3 Centrifugal pumps

As mentioned, the purchase cost of the centrifugal pumps includes the purchase cost of the pump structure as well as the purchase cost of the motor. The correlations for the base costs were obtained

from Seider et al.[16] equations 22.14 and 22.19, respectively. For both units, we assumed the purchase cost is the same as the base cost. For the pump structure this represents a single-stage cast iron unit and for the motor this represents a 3,600rpm open, drip-proof enclosure unit.

$$C^{j}_{p,pump\,structure} = \exp\left(9.717 - 0.6019 \times \ln S + 0.0519 \times (\ln S)^2\right) \quad \text{(S114)}$$

$$C^{j}_{p,motor} = \exp\left(5.8259 - 0.13141 \times \ln P_c + 0.053255 \times (\ln P_c)^2 + 0.28628 \times (\ln P_c)^3 - 0.0035549 \times (\ln P_c)^4\right) \quad \text{(S115)}$$

$$C^{j}_{p,pump} = C^{j}_{p,pump\,structure} + C^{j}_{p,motor} \quad \text{(S116)}$$

S4.5.4 Condenser

We assumed the condenser is a plate-and-frame exchanger. The correlation was obtained from Seider et al.[16] Table 22.32.

$$C_{p,condenser} = 8{,}880 A_{HX}^{0.42} \quad \text{(S117)}$$

S4.5.5 Vacuum pump

The vacuum pump is sized using the correlation for liquid-ring pumps from Seider et al.[16] Table 22.32.. Where $S_{vac}\left(\frac{ft^3}{min}\right)$ the volumetric flow rate entering the vacuum pump, this includes the leaked air as well as the fraction of the permeate stream that is not condensed in the vacuum system condenser. We developed simulations in Aspen Plus to determine this volume flow rate at varying leaked air values, W_{air}.

$$C_{p,\,vac\,pump} = 8{,}250 \times S_{vac}^{0.35} \quad \text{(S118)}$$

S4.6 Economic model – utilities

As shown in the main text in equation (46), an empirical expression was developed to determine the effect of utility costs based on the operating costs ($/yr) of the system. We need to determine $Heat$,

$Cool$ and $Elec$, which are the low-pressure steam, chilled water and electricity duties, respectively, in kW. These utilities were estimated using the following equations

$$Heat = \frac{1\,kW}{1000\,Watt} \times \sum \dot{Q}_{heat}^{k} \tag{S119}$$

$$Cool = \frac{1\,kW}{1000\,Watt} \times \dot{Q}_{cool} \tag{S120}$$

For electricity the consumers are centrifugal pumps and the vacuum pump. For the centrifugal pump only the motor power consumption, $P_c\ (Hp)$, needs to be considered. For the power needed to run the vacuum pump, $\dot{W}_{vac}\ (kW)$, we developed an empirical expression in Aspen Plus. Details on how we developed this expression can be found in Section S5.1. where vapor compress distillate of the flash tank to atmospheric conditions (100 kPa) with an isentropic compressor efficiency of 40%.

$$Power = \left(\sum P_c^k \times \frac{1\,kW}{0.7457\,Hp} + \dot{W}_{vac}\right) \tag{S121}$$

$$\dot{W}_{vac} = -2.48x10^{-4} \times W_{air}^2 + 0.45 \times W_{air} + 2.21 + 1.98 \times \ln(W_{air}) \tag{S122}$$

S4.7 BDO recovery

Recovery of BDO is defined as follow, where $\dot{m}_{perm}$ and $\dot{m}_{feed}$ are the mass flow rate of the composite permeate stream and overall feed to the system, respectively. As mentioned, the recovery of BDO in this process was >99.4% for all considered cases.

$$R = \left(1 - \frac{\dot{m}_{perm} w_{perm,BDO}}{\dot{m}_{feed} w_{feed,BDO}}\right) \times 100\% \tag{S123}$$

S4.8 Chemical engineering cost index

To account for the time value of money we used the following expression. $CE\ Cost\ Index$ is the Chemical Engineering (CE) cost index for a given year. For the SOT the CE_{SOT} value is 541/7, which corresponds to 2016[17], and the reference $CE_{reference}$value for Seider is 500[18].

$$New\ Installed\ Cost = \ Reference\ Installed\ Cost \times \left(\frac{CE\ Cost\ Index_{SOT}}{CE\ Cost\ Index_{Reference}}\right) \tag{S124}$$

S5. Correlations/Surrogates

Like the physical properties (density and viscosity) of the mixtures described in Section S2.3, some units in the process and some terms do not need to be exactly modeled. They can instead be obtained using correlations (or surrogates) that predict the output given an input. The correlations fit data collected from either a sensitivity analyses in Aspen Plus or from multiple runs of our previous rigorous SMB+distillation model. The data and correlations are available in Excel spreadsheets in the .zip folder of the Supplemental Material Data.

S5.1 Vacuum system correlations

The vacuum system was modeled in Aspen Plus, with the process flowsheet shown in **Figure** S6. The "AIR" stream represents the air leakage into the system calculated from Equation (S110) and "PERM" is the composite permeate stream of the membrane cascade. Following Seider et al., a flash tank (zero heat duty) was used to determine the water and BDO in equilibrium with air at the given temperature and pressure conditions. The vapor exiting the flash tank, "VAP", is compressed from vacuum to ambient pressure at a 40% isentropic efficiency, effectively approximating the vacuum pump unit, while "LIQ" represents the condensate. The main information extracted from this simulation is the vacuum pump, the volumetric flow rate of "VAP" (to cost the pump) and the quantity of water and BDO in "VAP" (to obtain the final mass and energy balances).

Since recovery is nearly constant (>99.5%) across all cases studied and the inlet and outlet BDO concentrations are fixed at 70wt% and 90wt%, respectively, the mass rate (6170 kg/hr) and composition (97.5 wt% water) of "PERM" remained constant throughout all Aspen Plus iterations. Pressure also remained constant at $P_p = 7\ kPa$, making mass rate (lb/hr) of "AIR" the only variable in this sensitivity analysis. Equations (S125) – (S126) show the correlations used to obtain vacuum pump work, $\dot{W}_{vac}$ (kW), the suction volumetric flow rate, $S_{vac}\left(\frac{ft^3}{min}\right)$nd the mass rate of water, $Water_{vac}\left(\frac{kg}{hr}\right)$, and BDO, $BDO_{vac}\left(\frac{kg}{hr}\right)$, in equilibrium with the leaked air.

$$Water_{vac} = -8.02x10^{-3} \times W_{air}^2 + 0.87 \times W_{air} + 5.66 + 3.98 \times \ln(W_{air}) \quad \text{(S125)}$$

$$BDO_{vac} = -1.79x10^{-4} \times W_{air}^{2} + 1.19x10^{-3} \times W_{air} + 9.61x10^{-3} - 1.33x10^{-3} \times \ln(W_{air}) \quad \text{(S126)}$$

The "AIR" mass rate ranged from 7 to 20 lb/hr, which was selected based on the results obtained in Figure 10 of the main text. Although varying "PERM" would improve the quality of the sensitivity analysis, the change is small across scenarios and keeping it constant should not significantly affect the validity of the obtained correlations. The data collected from Aspen Plus and details regarding the fitting process are available in the Supplemental Material.

S5.2 Total annualized cost correlation

As detailed in the main text, the total annualized cost (TAC) was represented as an empirical expression, which was derived from NREL's discount cash flow rate of return (DCFROR) model for biorefineries. This model incorporates financial terms, including equity, interest rates, depreciation, taxes, time value of money, and amortization, which are used to calculate the minimum fuel selling price (MFSP) of the fuel product ($/GGE, gallon of gasoline equivalent).

The total investment cost and annual operating costs of the biorefinery are approximately 700 $MM and 150 $MM/yr, respectively. Incorporating the cascade to the biorefinery thus amounts to slight changes in MFSP (<0.1 $/GGE), and economic metrics such TAC ($/yr) are better suited for analyzing the proposed design across multiple scenarios. This is also a more familiar metric that can be better interpreted by the reader.

Traditional estimates of TAC include calculating an annualized purchase equipment cost (C_p) and account for the cost impact of increasing utilities. Utilities includes the low-pressure steam (LPS) for the heat, the chilled water (CHW) for cooling and electricity ($POWER$). We also annualized the membrane purchase cost ($MEMB$). The multiplier for $Heat$ accounts for the imported fuel, boiler chemicals and expansion of the CHP capacity. The multiplier for $Cool$ reflects the rise in equipment size and electricity use for the refrigeration loop. For C_p, the overall coefficient includes a multiplier, a_1, that gives the total investment cost from the purchase equipment cost (typically the Lang factor) and an annualization term, a_2. We decided to develop an empirical expression for TAC, with the same

form as the typical TAC equation, where the obtained coefficients can be compared to values commonly used in techno-economic analyses.

$$TAC = \left[(a_1 \times a_2) \times C_p + b \times LPS + c \times CHW + d \times ELEC + e \times MEMB\right]\left(\frac{100}{R}\right)^f \qquad \text{(S127)}$$

The next step is determining TAC based on the output of the DCFROR model. As an underlying assumption we propose that a marginal increase in TAC must be equal to the marginal increase in revenue that result from adding equipment or increasing utilities. The assumption is grounded in the principle that, for any biorefinery to break even, any cost increase must be compensated by an increase in revenue. The increase in revenue is controlled by MFSP, which can then be readily used as a proxy for TAC and is reflected by the proposed relation.

$$\Delta TAC = \Delta Revenue \qquad \text{(S128)}$$

The biorefinery internally produces LPS and CHW and we estimated their multiplier by calculating the marginal increase in revenue that result from increasing one unit of these utilities. This procedure to estimate marginal costs aligns with the recommendations from the U.S. Department of Energy[19].

These multipliers reflect the complexity of producing these utility streams. For LPS this includes the imported natural gas and increase in size of the turbine and boiler. For CHW this includes the additional circulating cooling water and higher electricity demand of the refrigeration loop. This is a more realistic costing approach, as opposed to traditional methods where a unit import cost is often assumed for the utility streams despite being produced internally. As was mentioned in the main text, the coefficients obtained with this method closely agree with unit import costs reported in literature[20]. Lastly, we should also mention that we also evaluated other marginal increases (e.g. 10MW for heating and cooling and 100 kW for electricity), which yielded similar results. For membrane replacement we assume $e = \frac{0.2}{yr}$, which corresponds to a reasonable membrane replacement every five years and is based on existing pervaporation techno-economic studies[21, 22]. For the specific coated PVDF fibers used in this work, while exact quantification of membrane lifetime was not done, the authors have run pervaporation procedures to dewater organic/water mixtures for nearly 150 total hours on the same membrane module

with no measurable decrease in membrane permeate flux or change in separation performance that would indicate fouling. In addition, the vendor for the PVDF fibers reports that the membrane are designed to last up to 10 years for water treatment application, meaning the limiting factor is the coating[23].

$$b\left(\frac{\frac{\$}{yr}}{MW_{Heat}}\right) = \frac{Revenue\ (@LPS = 10MW) - Revenue\ (@LPS = 0MW)}{10\ MW} \tag{S129}$$

$$c\left(\frac{\frac{\$}{yr}}{MW_{Cool}}\right) = \frac{Revenue\ (@CHW = 1MW) - Revenue\ (@CHW = 0MW)}{1\ MW} \tag{S130}$$

$$d\left(\frac{\frac{\$}{yr}}{kW_{Power}}\right) = \frac{Revenue\ (@Power = 10kW) - Revenue\ (@Power = 0kW)}{10\ kW} \tag{S131}$$

$$f(-) = \frac{\ln\left[\frac{Revenue(@R = 100\%)}{Revenue(@R = 99.5\%)}\right]}{\ln\left(\frac{100}{99.5}\right)} \tag{S132}$$

We estimated the combined term ($a = a_1 \times a_2$) by calculating the incremental revenue from the added purchase equipment costs to the plant. Other marginal increases (e.g. 10 \$MM) were also considered and like the utility costs, this didn’t change the value of the multiplier.

$$a = (a_1 \times a_2)\left(\frac{\frac{\$}{yr}}{\$}\right) = \frac{Revenue\ \left(@C_p = 1\ \$MM\right) - Revenue\ \left(@C_p = 0\right)}{1\ \$MM} \tag{S133}$$

For equipment cost is a common practice to multiply C_p by a Lang factor to obtain the total investment cost and then apply a discount factor to spread it over a project’s lifetime. However, using a standard Lang factor may fail to capture the specific characteristics of this biorefinery. The term a_1 would then be the Lang factor, which in the original biorefinery design is 3.8.

Additionally, only accounting for the discount factor neglects other economic elements from the cost model such corporate taxes, equity, depreciation schemes, and increased labor, maintenance, and overhead expenses. The term a_2 is thus generally obtained from the cost recovery factor in the annuity equation for present value, where i is the interest rate and N is the number of years.

$$a_2 = \frac{i(1+i)^N}{(1+i)^N - 1} \tag{S134}$$

Using the internal rate of return ($i = 0.1$) and the plant lifetime ($N = 30$) from the biorefinery report, gives $a_2 = 0.106$. This gives $a = (a_1 \times a_2) = 0.403$, which is comparable to the value of 0.29 obtained through our proposed methodology. The obtained multiplier of 0.29 thus effectively serves as an amortization factor that accurately accounts for the economic model complexities and is comparable to the value obtained from more common methods. This value captures more realistically the intricacies of the financial model, which would otherwise be ignored if only the annuity term was considered. **Table S10** summarizes the obtained multipliers.

For equations (S129) – (S133), all baseline revenues, $Revenue\ (@LPS = 0)$, $Revenue\ (@CHW = 0)$, $Revenue\ (@Power = 0)$, $Revenue\ (@R = 100)$ and $Revenue\ \left(@C_p = 0\right)$ are base revenue. This is the revenue obtained prior to the incorporation of a pervaporation cascade.

S6. Markov chain Monte Carlo hyperparameters and results

The values of the entries in the proposal width vector, w_i, were determined via fine-tuning. **Table S11** shows the values.

The covariance matrix of the parameters is one of the main results of the MCMC solution. **Table S12** shows the entries of this matrix, units are given in Equation $(\mathrm{S}42)$.

S7. Process design recommendation

As mentioned in the main manuscript, based on the results obtained across the various scenarios, values of $A_{max,memb} = 3200\ m^2$ and $T_{feed} = 363\ K$ were selected. An optimal base case was then developed as a final process design recommendation. In the following sections we present the details of this final design. The mean permeance parameter obtained from the MCMC, $\bar{\boldsymbol{\theta}}_{\boldsymbol{MCMC}}$, was used to develop the proposed process design.

S7.1 Mass and energy balance results

The final recommendation for the cascade process design is presented in **Figure S7**. The design consists of 10 pervaporation stages, for which the mass and energy balances were obtained by solving the steps shown in Algorithm S2. **Table S13** shows the details of the mass and energy balances for each stream and **Table S14** shows the details of the membrane modules.

S7.2 Sensitivity analysis

We also considered the effect of final BDO purity on the main process metrics of this design. Although a value of 90% was chosen for the case study in the main text, in practice this value can be adjusted for the downstream upgrading step. **Figure S8** shows how the number of membranes, membrane area, heat exchanger area, BDO recovery, total annualized cost, operating expenses, capital expenses and membrane costs vary with BDO purity values between 70% – 99.9%.

As expected, as the BDO purity requirement increases the proposed cascade requires additional stages. This leads to higher membrane and heat exchanger areas, and therefore higher costs (capital and operating). Furthermore, BDO recovery decreases with increased required BDO purity. The reason is because more permeate needs to be produced in order to remove the water content, which leads to more BDO losses. Although a comprehensive analysis of the process economics was beyond the scope of this work, it is important to understand the impact of these variables and this sensitivity analysis provides an efficient representation of the influence of the extent of separation of key metrics.

S8. Supplemental Tables

Table S1. Control experiment of the dense PVDF membrane (Pp = 1.08 kPa)

Temperature (K)	Water in feed (%)	Flux (LMH)
353	100	0.3

Table S2. BDO rejection, pure water permeate flux at 353.15 K, and permeate flux using an 80% BDO/20% water mixture at 353.15 K as a function of number of coating layers using the coating procedure detailed in the main text.

Number of coating layers	BDO Rejection (%)	Pure water permeate flux $(L\,m^{-2}\,h^{-1})$	BDO (80) / water (20wt%) permeate flux $(L\,m^{-2}\,h^{-1})$
c	10	–	6.7
1	68	0.71	0.38
2	80	0.54	0.26
3	89	0.51	0.28
4	96	–	0.32

Table S3. UNIQUAC model structural pure component parameters for BDO and water.

**Component**	r_i	q_i
**BDO**	3.756	3.32
**Water**	0.92	1.4

Table S4. UNIQUAC binary interaction coefficients a_{ij} for BDO and water. Self-interaction coefficients (a_{ii}) are zero.

**Comp.** i **/ Comp.** j	$a_{ij}\left(\frac{cal}{mol}\right)$	$a_{ji}\left(\frac{cal}{mol}\right)$
**BDO/Water**	1098	-581

Table S5. Reference enthalpy and heat capacity values at constant pressure. T_{ref} is 70°C.

Component	H_{ref}^{vap}	H_{ref}^{liq}	C_p^{vap}	C_p^{liq}
**BDO**	-476	-537	141	195
**Water**	-240	-282	33.8	78.2

Table S6. Antoine constants, $log_{10}(P) = A - \frac{B}{T+C}$.

Component	A	$B(K)$	$C(K)$
**BDO**	6.07	2616.75	-24.57
**Water**	4.65	1435.26	-64.85

Table S7. Molar density coefficients, pure component

Component	$\rho_i^0 \left(\frac{kmol}{m^3}\right)$	T^0 (K)	β_i x 10^4 (K^{-1})
**BDO**	10.29	363	11.42
**Water**	53.46	363	6.028

Table S8. Molar density coefficients, mixture

Coefficient	μ^0 x 10^5 $(kg\,m^{-1}\,s^{-1})$	T^0 (K)	A	B (K)
**Value**	9.36	297.1	3.19	72.53

Table S9. Initial guesses and upper and lower bounds for parameter estimation

**Value**	$Q_{0,water} \times 10^8$ $(mol\,m^{-2}\,s^{-1}\,Pa^{-1})$	$Q_{0,BDO} \times 10^8$ $(mol\,m^{-2}\,s^{-1}\,Pa^{-1})$	$E_{a,water}$ $(kJ\,mol^{-1})$	$A_{conc,BDO}$ $(-)$
**Initial Guess**	9.67	1.00	-10.10	2.68
**Upper Bound**	100.0	10.0	100	10
**Lower Bound**	0.1	0.01	-100	-10

Table S10. Multipliers for Equation (S127) and unit energy normalized values. We assumed 7880 operating hours per year. Where applicable, values in parentheses in the "Normalized" column are unit energy costs obtained from literature.

Multiplier	$Value$	$Normalized$
**a = a$_1$ x a$_2$**	$0.29\left(\frac{\frac{\$}{yr}}{\$}\right)$	N/A
**b**	$86{,}317\left(\frac{\frac{\$}{yr}}{MW_{Heat}}\right)$	$3.04\left(\frac{\$}{GJ_{LPS}}\right)$ ($7.78\,\frac{\$}{GJ_{LPS}}$, Kiss et al 2013[20])
**c**	$117{,}578\left(\frac{\frac{\$}{yr}}{MW_{Cool}}\right)$	$4.14\left(\frac{\$}{GJ_{CHW}}\right)$ ($4.43\,\frac{\$}{GJ_{CHW}}$, Kiss et al 2013[20])
**d**	$561\left(\frac{\frac{\$}{yr}}{kW_{Power}}\right)$	$0.071\left(\frac{\$}{kWh_{Elec.}}\right)$ ($0.068\,\frac{\$}{kWh_{Elec.}}$, Davis et al 2018[17])
**e**	$0.20\left(\frac{\frac{\$}{yr}}{\$}\right)$	N/A
**f**	0.85 (-)	N/A

N/A: Not applicable, coefficient is not related to an energy duty so cannot be normalized to a unit energy cost.

Table S11. Proposal width values, where $w_i = \left[w_{Q_{0,water}}, w_{Q_{0,BDO}}, w_{E_{A,water}}, w_{A_{conc,BDO}}\right]$.

w_i	$Q_{0,water} \times 10^8$ $(mol\,s^{-1}\,m^{-2}\,Pa)$	$Q_{0,BDO} \times 10^8$ $(mol\,s^{-1}\,m^{-2}\,Pa)$	$E_{A,water}$ $(kJ\,mol^{-1})$	$A_{conc,BDO}$ $(-)$
**Value**	0.1	0.05	0.4	0.15

Table S12. Covariance Cov_{MCM} matrix of the permeance parameters.

	$Q_{0,water}$	$Q_{0,BDO}$	$E_{A,water}$	$A_{conc,BDO}$
$Q_{0,water}$	4.79×10^{-18}	5.58×10^{-19}	-4.62×10^{-6}	-1.83×10^{-11}
$Q_{0,BDO}$	5.58×10^{-19}	6.88×10^{-18}	9.80×10^{-7}	-1.41×10^{-9}
$E_{A,water}$	-4.62	9.80×10^{-7}	1.15×10^{7}	-1.78×10^{2}

$\boldsymbol{A_{conc,BDO}}$	$1.83\times\ 10^{-11}$	$-1.41\times\ 10^{-9}$	$-1.78\times\ 10^{2}$	$3.43\times\ 10^{-1}$

Table S13. Aspen Plus process flowsheet detailed mass balance

**Stream Number**		1	2	3	4	5	6	7	8	9	10
From			PUMP1	HX1	MEMB1	MEMB1	PUMP2	HX2	MEMB2	MEMB2	PUMP3
To		PUMP1	HX1	MEMB1	MIXER	PUMP2	HX2	MEMB2	MIXER	PUMP3	HX3
Temperature	K	363	363	363	312	344	344	363	312	343	343
Pressure	Pa	20265	101325	101325	7093	91520	101325	101325	7093	94637	101325
Vapor Fraction		0	0	0	1	0	0	0	1	0	0
Mass Flow (Total)	kg/hr	27000	27000	27000	671	26329	26329	26329	666	25663	25663
BDO	kg/hr	18900	18900	18900	17	18883	18883	18883	17	18866	18866
WATER	kg/hr	8100	8100	8100	654	7446	7446	7446	649	6797	6797

Table S13. Aspen Plus process flowsheet detailed mass balance

**Stream Number**		11	12	13	14	15	16	17	18	19	20
From		HX3	MEMB3	MEMB3	PUMP4	HX4	MEMB4	MEMB4	PUMP5	HX5	MEMB5
To		MEMB3	MIXER	PUMP4	HX4	MEMB4	MIXER	PUMP5	HX5	MEMB5	MIXER
Temperature	K	363	312	343	343	363	312	342	342	363	312
Pressure	Pa	101325	7093	94326	101325	101325	7093	93946	101325	101325	7093
Vapor Fraction		0	1	0	0	0	1	0	0	0	1
Mass Flow (Total)	kg/hr	25663	660	25003	25003	25003	653	24350	24350	24350	646
BDO	kg/hr	18866	17	18849	18849	18849	16	18833	18833	18833	16
WATER	kg/hr	6797	643	6154	6154	6154	636	5518	5518	5518	630

Table S13. Aspen Plus process flowsheet detailed mass balance

**Stream Number**		21	22	23	24	25	26	27	28	29	30
From		MEMB5	PUMP6	HX6	MEMB6	MEMB6	PUMP7	HX7	MEMB7	MEMB7	PUMP8
To		PUMP6	HX6	MEMB6	MIXER	PUMP7	HX7	MEMB7	MIXER	PUMP8	HX8
Temperature	K	341	341	363	312	341	341	363	312	340	340
Pressure	Pa	93475	101325	101325	7093	92884	101325	101325	7093	92130	101325
Vapor Fraction		0	0	0	1	0	0	0	1	0	0
Mass Flow (Total)	kg/hr	23705	23705	23705	638	23066	23066	23066	631	22435	22435
BDO	kg/hr	18817	18817	18817	15	18802	18802	18802	15	18787	18787
WATER	kg/hr	4888	4888	4888	623	4265	4265	4265	616	3649	3649

Table S13. Aspen Plus process flowsheet detailed mass balance

**Stream Number**		31	32	33	34	35	36	37	38	39	40
From		HX8	MEMB8	MEMB8	PUMP9	HX9	MEMB9	MEMB9	PUMP10	HX10	MEMB10
To		MEMB8	MIXER	PUMP9	HX9	MEMB9	MIXER	PUMP10	HX10	MEMB10	MIXER
Temperature	K	363	312	339	339	363	312	338	338	363	312
Pressure	Pa	101325	7093	91147	101325	101325	7093	89843	101325	101325	7093
Vapor Fraction		0	1	0	0	0	1	0	0	0	1
Mass Flow (Total)	kg/hr	22435	622	21813	21813	21813	614	21199	21199	21199	369
BDO	kg/hr	18787	15	18772	18772	18772	14	18758	18758	18758	10
WATER	kg/hr	3649	608	3041	3041	3041	599	2441	2441	2441	359

Table S13. Aspen Plus process flowsheet detailed mass balance

**Stream Number**		41	42	43	44	45	46	47
From		MEMB10	PUMP FINAL	HX FINAL	MIXER	VAC COND	VAC COND	PUMP COND
To		PUMP11	HX FINAL		VAC COND		PUMP COND	
Temperature	K	348	352	523	312	312	312	312
Pressure	Pa	74769	4559625	4559625	7093	7093	7093	101325
Vapor Fraction		0	0	0	1	1	0	0
Mass Flow (Total)	kg/hr	20830	20830	20830	6170	258	5912	5912
BDO	kg/hr	18748	18748	18748	152	0	152	152
WATER	kg/hr	2082	2082	2082	6018	258	5760	5760

Table S14. Dimensions and specification of the membrane modules of the final design recommendation (10 pervaporation stages). As mentioned in the main text each of these membrane modules consists of submodules arranged in parallel. Around 120,000 fibers are packed per module.

**Parameter**	**Value**
**Membrane fibers**	
Fiber diameter (mm)	0.85
Fiber length (m)	2.5
**Membrane module and submodules (stages 1 – 9)**	
Area per membrane module ($\frac{m^2}{module}$)	3200
Number of submodules	4
Area per submodule ($\frac{m^2}{submodule}$)	800
Module diameter (cm)	40.2
**Membrane module and submodules (stages 10)**	
Area per membrane module ($\frac{m^2}{module}$)	1580
Number of submodules	2
Area per submodule ($\frac{m^2}{submodule}$)	790
Module diameter (cm)	40.0

S9. Supplemental Figures

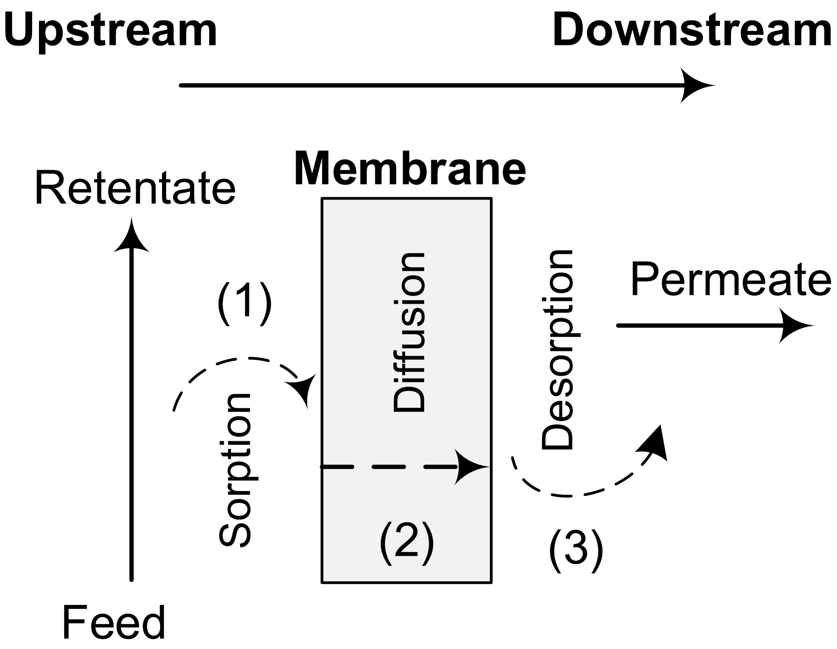

Figure S1. Representation of the pervaporation mechanism, illustrating the sequential steps: (1) sorption at the membrane interface on the upstream (feed/retentate) side, (2) diffusion through the membrane, and (3) desorption on the downstream (permeate) side.

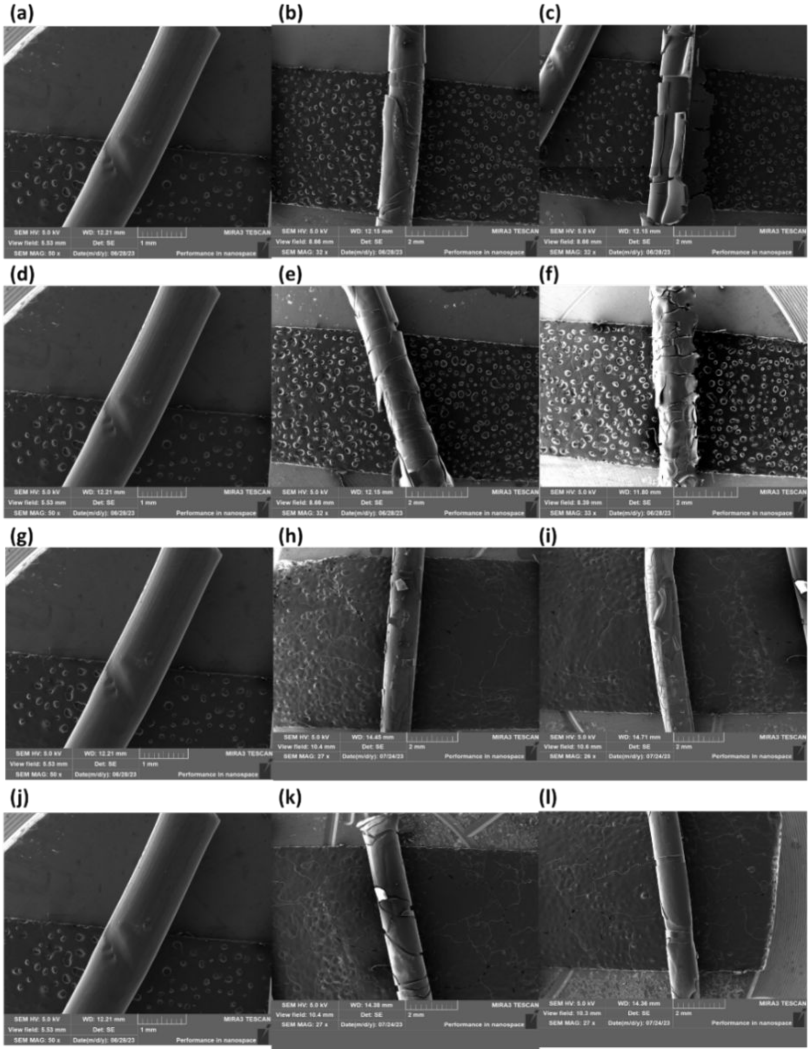

Figure S2. Dip coated PVDF fibers after the indicated dip coating procedure: (a, d, g, j) porous pretreated PVDF fiber; (b) 20 ml of PVDF dispersion, 0.2g of Easaqua 30s of dipping time; (c) 20 ml of PVDF, 0.2g of Easaqua 120s of dipping time; (e) 20 ml of PVDF dispersion, 0.4g of Easaqua 30s of dipping time; (f) 20 ml of PVDF dispersion, 0.4g of Easaqua 120s of dipping time; (h) 20 ml of PVDF dispersion, 0.4g of Easaqua 10s of dipping time; (i) 20 ml of PVDF dispersion, 0.2g of Easaqua 10s of dipping time; (k) 20 ml of PVDF dispersion, 0.4g of Easaqua 10s of dipping time; (l) 20 ml of PVDF dispersion solution, 0.4g of Easaqua 5s of dipping time.

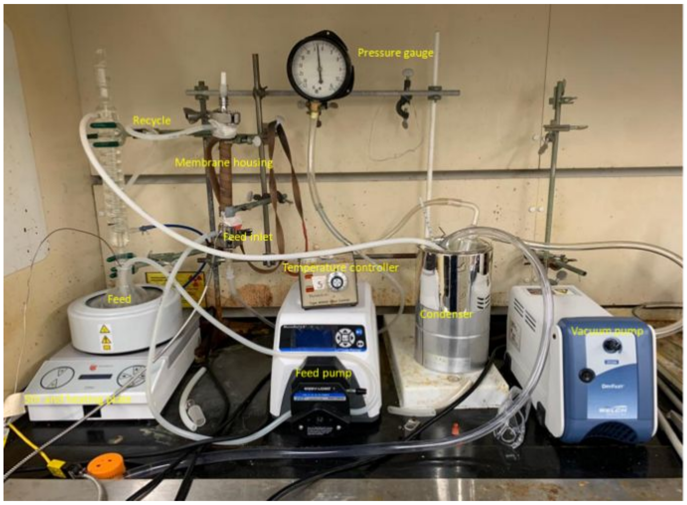

Figure S3. Pervaporation setup used for BDO rejection experiment from a simulated BDO-rich SMB+distillation separation product.

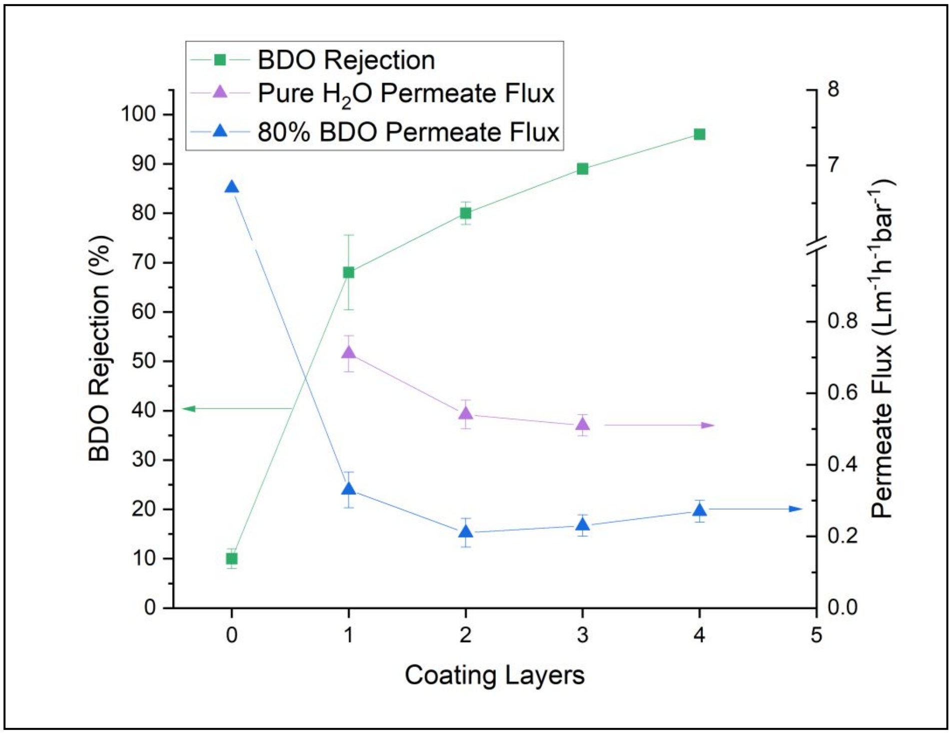

Figure S4. BDO rejection (green points), pure water permeate flux at 353.15 K (purple points), and permeate flux using an 80% BDO/20 wt% water mixture at 353.15 K (blue points) as a function of number of coating layers using the coating procedure detailed in the main text.

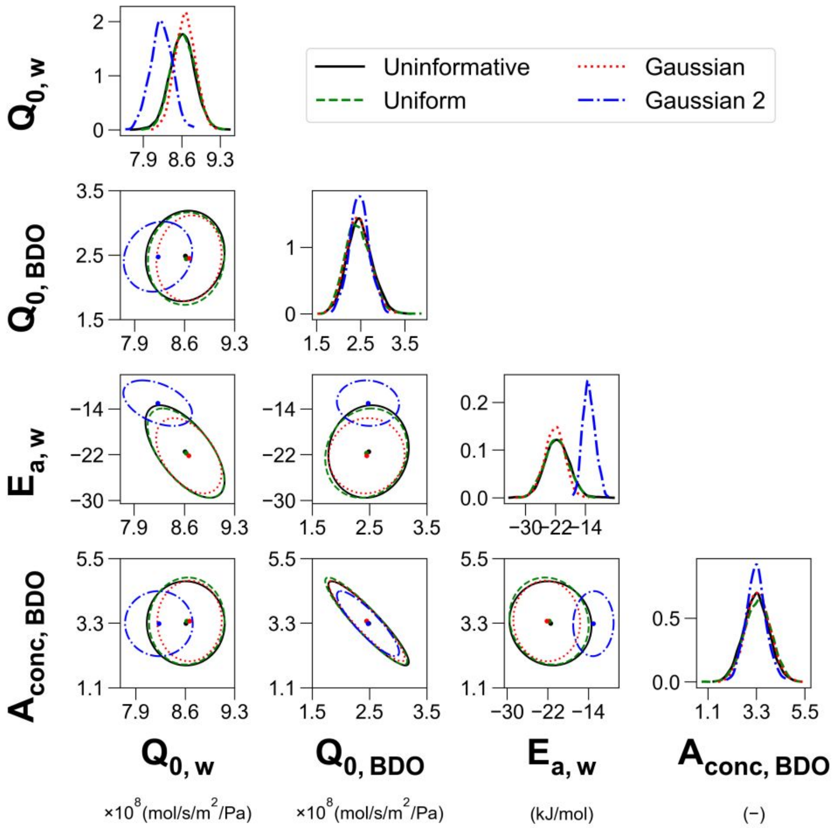

Figure S5. Marginal and joint posterior distributions of the model parameters, $\boldsymbol{\theta}$, under different prior distributions, $\boldsymbol{p}(\boldsymbol{\theta})$.

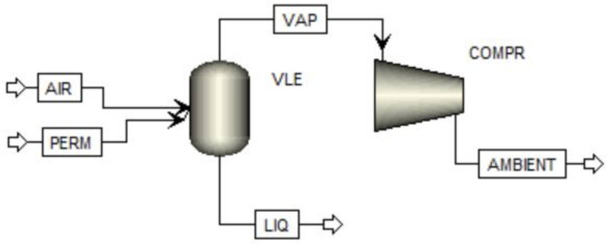

Figure S6. Diagram of the vacuum system

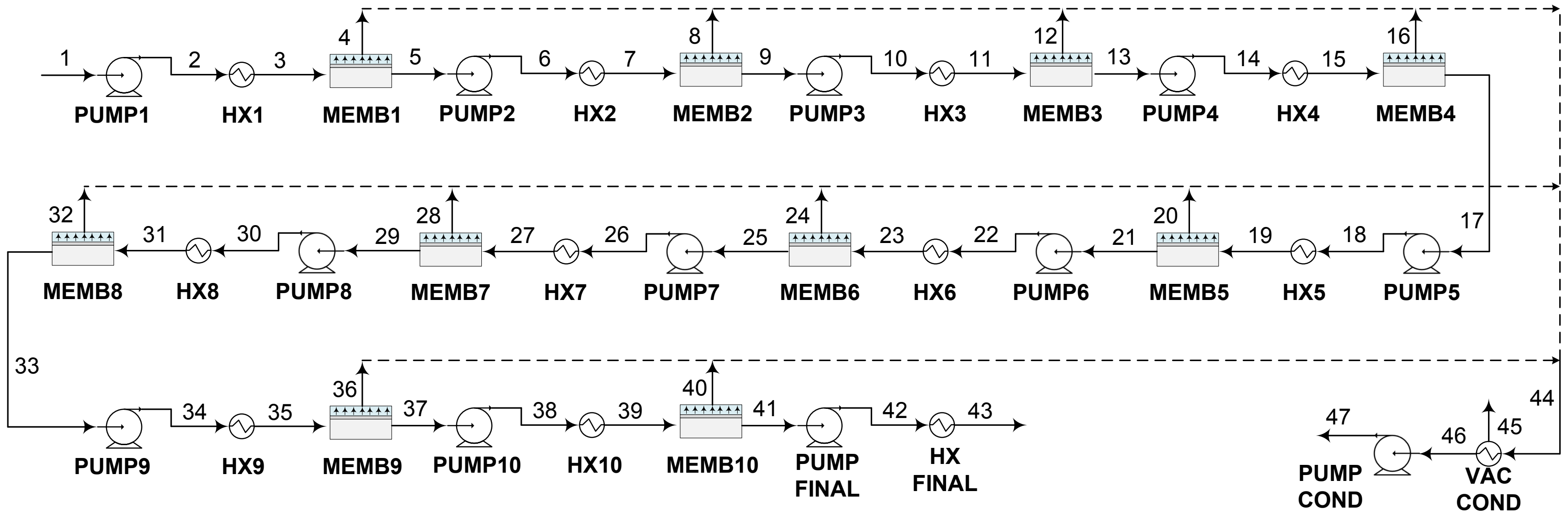

Figure S7. Detailed process flowsheet diagram of the proposed cascade pervaporation. Details of the mass and energy balance of each stream can be found in **Table S13**

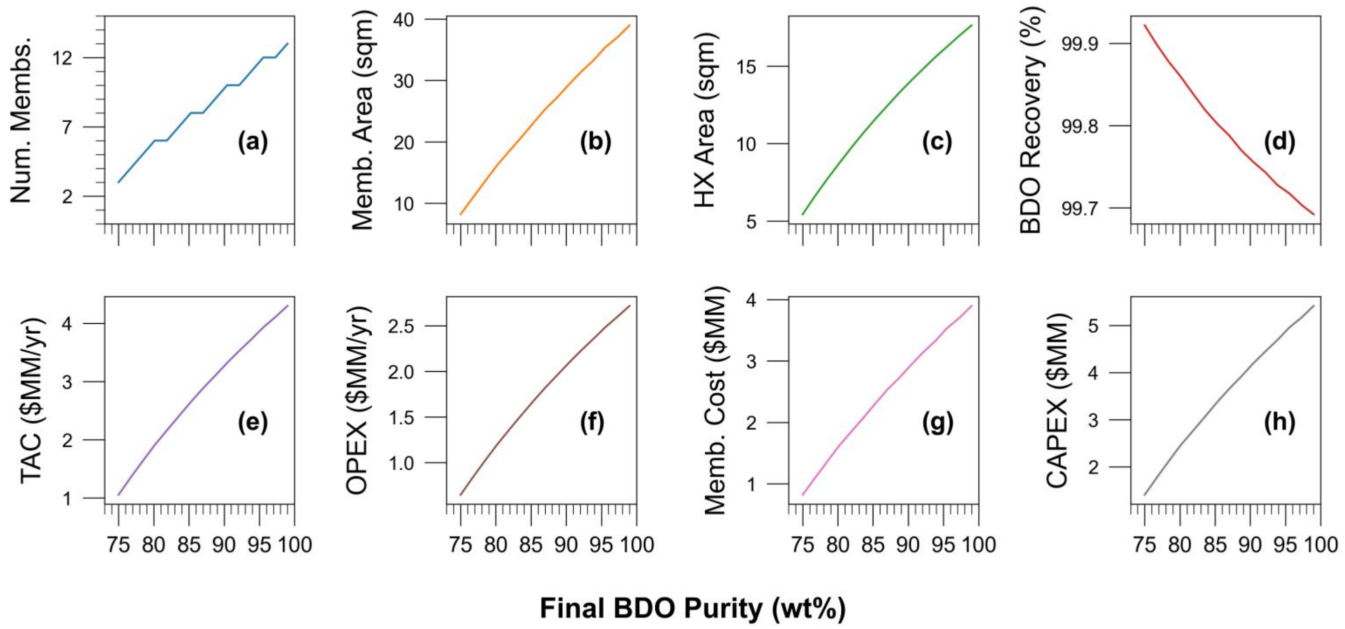

Figure S8. Effect of final BDO purity on (a) number of membranes, (b) membrane area, (c) heat exchanger area, (d) BDO recovery, (e) total annualized cost, (f) operating expenses, (g) membrane cost, and (h) capital expenses

References

(S1) Aspen Properties® Aspen Technology Inc. Bedford, MA, USA, 2022.

(S2) Linstrom, P.J and Mallard, W.G.. NIST Chemistry WebBook, NIST Standard Reference Database Number 69, National Institute of Standards and Technology, Gaithersburg MD, 20899.

(S3) Velho, P.; Oliveira, R. A.; Macedo, E. A. Correlating Excess Volumes for Binary Mixtures of Green Solvents with the Help of Density Functional Theory. *Industrial & Engineering Chemistry Research* **2024**, *63* (36), 15990-15998. DOI: 10.1021/acs.iecr.4c02413.

(S4) van Velzen, D.; Cardozo, R. L.; Langenkamp, H. A Liquid Viscosity-Temperature-Chemical Constitution Relation for Organic Compounds. *Industrial & Engineering Chemistry Fundamentals* **1972**, *11* (1), 20-25. DOI: 10.1021/i160041a004.

(S5) Bowman, C.; Larson, K.; Roitershtein, A.; Stein, D.; Matzavinos, A. Bayesian Uncertainty Quantification for Particle-Based Simulation of Lipid Bilayer Membranes: Modeling and Applications. 2018; pp 77-102.

(S6) Kawajiri, Y. Model-based optimization strategies for chromatographic processes: a review. *Adsorption-Journal of the International Adsorption Society* **2021**, *27* (1), 1-26. DOI: 10.1007/s10450-020-00251-2.

(S7) Kalyanaraman, J.; Kawajiri, Y.; Realff, M. J. Bayesian Estimation, Uncertainty Propagation and Design of Experiments for CO Adsorption on Amine Sorbents. *Proceedings of the 8th International Conference on Foundations of Computer-Aided Process Design* **2014**, *34*, 345-350.

(S8) Kalyanaraman, J.; Fan, Y. F.; Labreche, Y.; Lively, R. P.; Kawajiri, Y.; Realff, M. J. Bayesian estimation of parametric uncertainties, quantification and reduction using optimal design of experiments for CO adsorption on amine sorbents. *Computers & Chemical Engineering* **2015**, *81*, 376-388. DOI: 10.1016/j.compchemeng.2015.04.028.

(S9) Kalyanaraman, J.; Kawajiri, Y.; Lively, R. P.; Realff, M. J. Uncertainty Quantification via Bayesian Inference Using Sequential Monte Carlo Methods for CO Adsorption Process. *Aiche Journal* **2016**, *62* (9), 3352-3368. DOI: 10.1002/aic.15381.

(S10) Kalyanaraman, J.; Kawajiri, Y.; Realff, M. J. Bayesian design of experiments for adsorption isotherm modeling. *Computers & Chemical Engineering* **2020**, *135*. DOI: ARTN 106774 10.1016/j.compchemeng.2020.106774.

(S11) Yamamoto, Y.; Yajima, T.; Kawajiri, Y. Uncertainty quantification for chromatography model parameters by Bayesian inference using sequential Monte Carlo method. *Chemical Engineering Research & Design* **2021**, *175*, 223-237. DOI: 10.1016/j.cherd.2021.09.003.

(S12) Sugiyama, H.; Yamamoto, Y.; Suzuki, K.; Yajima, T.; Kawajiri, Y. Parameter estimation for reactive chromatography model by Bayesian inference and parallel sequential Monte Carlo. *Chemical Engineering Research & Design* **2024**, *203*, 378-390. DOI: 10.1016/j.cherd.2024.01.056.

(S13) *Multistart Solver — Pyomo 6.8.0 Documentation*. 2024. https://pyomo.readthedocs.io/en/6.8.0/contributed_packages/multistart.html (accessed 2025-05-30).

(S14) Constantino, D.; Faria, R.; Ribeiro, A.; Rodrigues, A. Process re-intensification strategy for butyl acrylate manufacturing: Enhancement, scaling-up and economical evaluation. *Journal of Advanced Manufacturing and Processing* **2020**, *2*. DOI: 10.1002/amp2.10048.

(S15) Marco Avendano, Q. F., Jianpei Lao, Sankar Nair, Matthew J Realff. Simultaneous optimization of simulated moving bed adsorption and distillation for 2,3-butanediol recovery. *Aiche Journal* **2024**.

(S16) Seider, W. D.; Seader, J. D.; Lewin, D. R. *Product and Process Design Principles: Synthesis, Analysis and Design*; Wiley, 2008.

(S17) Davis, R. E.; Grundl, N. J.; Tao, L.; Biddy, M. J.; Tan, E. C.; Beckham, G. T.; Humbird, D.; Thompson, D. N.; Roni, M. S. *Process Design and Economics for the Conversion of Lignocellulosic Biomass to Hydrocarbon Fuels and Coproducts: 2018 Biochemical Design Case Update; Biochemical Deconstruction and Conversion of Biomass to Fuels and Products via Integrated Biorefinery Pathways*; United States, 2018. DOI: 10.2172/1483234.

(S18) Seider, W. D.; Lewin, D. R.; Seader, J. D.; Widagdo, S.; Gani, R.; Ng, K. M. *Product and Process Design Principles: Synthesis, Analysis and Evaluation*; Wiley, 2016.

(S19) *How to Calculate the True Cost of Steam*; U.S. Department of Energy, Energy Efficiency and Renewable Energy, Washington, DC, 2003. https://www.energy.gov/eere/amo/articles/how-calculate-true-cost-steam (accessed November, 2024).

(S20) Kiss, A. A. Design, Control and Economics of Distillation. *Advanced Distillation Technologies: Design, Control and Applications* **2013**, 37-65. DOI: 10.1002/9781118543702.

(S21) Prihatiningtyas, I.; Hussien Al-Kebsi, A. A.; Hartanto, Y.; Zewdie, T. M.; Van der Bruggen, B. Techno-economic assessment of pervaporation desalination of hypersaline water. *Desalination* **2022**, *527*. DOI: 10.1016/j.desal.2021.115538.

(S22) O'Brien, D. J.; Roth, L. H.; McAloon, A. J. Ethanol production by continuous fermentation-pervaporation: a preliminary economic analysis. *Journal of Membrane Science* **2000**, *166* (1), 105-111. DOI: Doi 10.1016/S0376-7388(99)00255-0.

(S23) Arkema. *Kynar® PVDF membrane ultrafiltration of Toulouse wastewater: reliability and permanence of efficiency*. 2021. https://www.arkema.com/global/en/resources/post/hpp/kynar-pvdf-membrane-ultrafiltration-of-toulouse-wastewater/ (accessed 2025-05-30).
